# *De novo* design of RNA-binding proteins with a prion-like domain related to ALS/FTD proteinopathies

**DOI:** 10.1038/s41598-017-17209-0

**Published:** 2017-12-04

**Authors:** Kana Mitsuhashi, Daisuke Ito, Kyoko Mashima, Munenori Oyama, Shinichi Takahashi, Norihiro Suzuki

**Affiliations:** Departments of Neurology, Keio University School of Medicine, 35 Shinanomachi, Shinjuku-ku, Tokyo, 160-8582 Japan

**Keywords:** Amyotrophic lateral sclerosis, Neurodegeneration

## Abstract

Aberrant RNA-binding proteins form the core of the neurodegeneration cascade in spectrums of disease, such as amyotrophic lateral sclerosis (ALS)/frontotemporal dementia (FTD). Six ALS-related molecules, TDP-43, FUS, TAF15, EWSR1, heterogeneous nuclear (hn)RNPA1 and hnRNPA2 are RNA-binding proteins containing candidate mutations identified in ALS patients and those share several common features, including harboring an aggregation-prone prion-like domain (PrLD) containing a glycine/serine-tyrosine-glycine/serine (G/S-Y-G/S)-motif-enriched low-complexity sequence and rich in glutamine and/or asparagine. Additinally, these six molecules are components of RNA granules involved in RNA quality control and become mislocated from the nucleus to form cytoplasmic inclusion bodies (IBs) in the ALS/FTD-affected brain. To reveal the essential mechanisms involved in ALS/FTD-related cytotoxicity associated with RNA-binding proteins containing PrLDs, we designed artificial RNA-binding proteins harboring G**/**S-Y-G**/**S-motif repeats with and without enriched glutamine residues and nuclear-import/export-signal sequences and examined their cytotoxicity *in vitro*. These proteins recapitulated features of ALS-linked molecules, including insoluble aggregation, formation of cytoplasmic IBs and components of RNA granules, and cytotoxicity instigation. These findings indicated that these artificial RNA-binding proteins mimicked features of ALS-linked molecules and allowed the study of mechanisms associated with gain of toxic functions related to ALS/FTD pathogenesis.

## Introduction

Growing evidence indicates that disturbed RNA quality control systems mediated by aberrant RNA-binding proteins, such as TDP-43 and FUS, form the core of the neurodegeneration cascade in ALS/FTD^1^. Six ALS-related molecules, TDP-43, FUS, TAF15, EWSR1, heterogeneous nuclear (hn) RNPA1 and hnRNPA2 are RNA-binding proteins containing candidate mutations identified in ALS patients. These molecules share several common features. First, they harbor a prion-like domain (PrLD) and are aggregation-prone^2^, and PrLDs involving self-propagating amyloid-fibril conformations exhibit two critical features: they contain a glycine/serine-tyrosine-glycine/serine (G/S-Y-G/S)-motif-enriched low-complexity sequence (regions of protein sequences with biased amino acid composition)^3^ and are rich in glutamine and/or asparagine (Q/N)^2,4^. PrLDs can mediate pathologically aggregated states observed in the ALS/FTD brain as a result of aging, and/or ALS-linked mutations clustered in the PrLDs of TDP-43 and FUS and which accelerate this toxic conversion to initiate neurodegeneration.

Second, they are included as components of RNA or messenger ribonucleoprotein granules, such as stress granules (SGs), which are involved in the RNA quality control system by stabilizing and storing mRNAs and arresting translation to prevent the accumulation of misfolded proteins^5–7^. A low-complexity sequence in PrLDs mediates interactions between biophysical molecules and RNA granules and it drives the assembly of cytoplasmic RNA granules, with ALS-linked mutations in TDP-43, FUS, and hnRNPA2^8–11^.

Third, a common pathological feature of ALS/FTD is the mislocalization of candidate RNA-binding proteins from the nucleus to the cytoplasm and their subsequent formation of cytoplasmic inclusion bodies (IBs) in disease-affected regions. In FUS, ALS-associated mutations are clustered at the non-classical nuclear-localization signal (NLS) in the C-terminus, which directly impedes nuclear localization^10,12–14^. The mutation of a truncated protein lacking a NLS (R495X) was identified as being causative of juvenile ALS with rapid disease progression, indicating that impairment of FUS nucleocytoplasmic trafficking is directly linked to motor-neuron degeneration^15^. Given these commonalities, cytoplasmic aggregation of RNA-binding proteins could constitute a critical process in the development of the ALS/FTD pathological cascade linked to the RNA quality control system.

An emerging question involving these ALS/FTD-related RNA-binding proteins is whether their associated cytotoxicity is dependent upon specific physiological functions related to target RNAs or their robust and non-specific assembly with ribonucleoproteins. To address this, we generated artificial RNA-binding proteins harboring repeats of the G**/**S-Y-G**/**S-motif-enriched low-complexity sequence with and without enriched glutamine residues and signal sequences for nuclear import/export. To minimize superfluous functions in mammalian cells, a well-characterized plant-based RNA-recognition domain [poly(A)-binding protein (PABPs)]^16^ was selected as a standard RNA-recognition motif (RRM). These proteins exhibited features similar to those of ALS-linked molecules, including insoluble aggregation, formation of cytoplasmic IBs and RNA-granule components, and instigation of cytotoxicity. These findings indicated that cytoplasmic RNA-binding proteins containing PrLDs can initiate neurotoxicity independent of their specific physiological functions. We propose that these artificial RNA-binding proteins effectively replicated features of ALS-linked molecules and constitute novel experimental molecules that enable enhanced understanding of the molecular basis of ALS/FTD-related cytotoxicity.

## Results

### Characterization of SYG and SYGQ-nuclear-export signal (NES)-green fluorescent protein (GFP) expression in cultured cells

**Figure 1:**
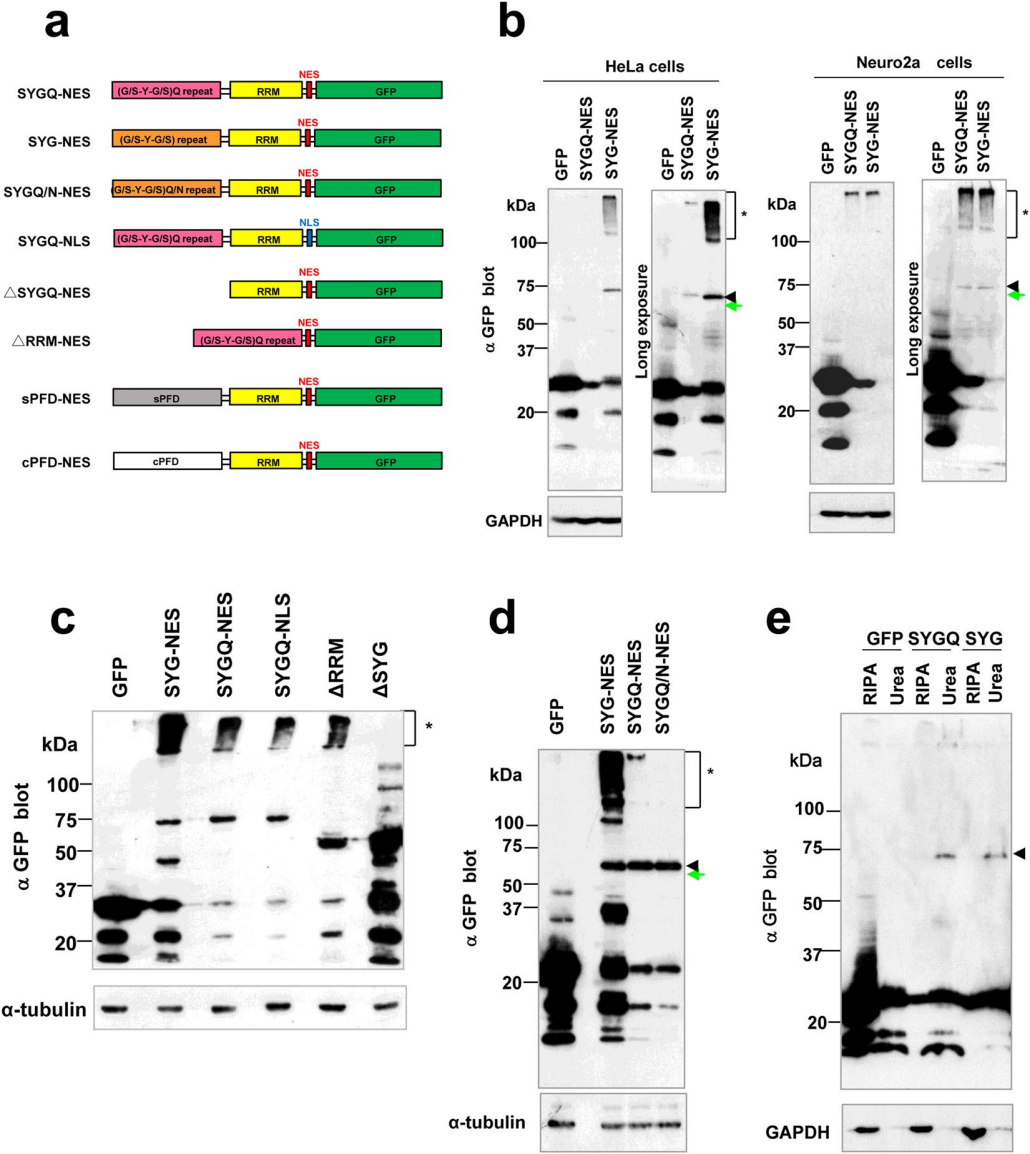
Expression in cultured mammalian cells of artificial RNA-binding proteins containing PrLDs. (**a**) Schematic diagram showing artificial RNA-binding proteins containing PrLDs, as well as the deletion constructs. All constructs contained a GFP tag at the C-terminus. sPFD: synthetic prion-forming domain; cPFD: control prion-forming domain [exhibiting low degrees of prion propensity^17^]. (**b**) Immunoblot analysis of SYG/SYGQ-NES expression in transfected HeLa and Neuro2a cells. Lysates of cells transiently transfected with plasmids were fractionated by SDS-PAGE and analyzed by immunoblot using a mouse monoclonal anti-GFP antibody. α-tubulin was used as an internal control. Asterisks (*) indicate aggregation resolved using stacking gels. Arrowheads indicate full-length SYG/SYGQ-NES. Green arrows indicate predicted size. (**c**) Immunoblot analysis of deletion constructs expressed in 293 T cells. Protein (12 μg/lane) was loaded on a 4% to 20% Tris-glycine gradient gel and detected using an anti-GFP antibody for western blot analysis. Asterisks (*) indicate aggregation resolved using stacking gels. (**d**) Immunoblot analysis of SYG, SYGQ, and SYGQ/N-NES expression in transfected 293 T cells. α-tubulin was used as an internal control. Asterisks (*) indicate aggregation, and arrowheads indicate full-length proteins. (**e**) Neuro2a cell lysates expressed of SYG/SYGQ-NES were separated into RIPA-soluble and -insoluble urea fractions. Full-length proteins (indicated by arrowheads) were detected in only the insoluble fractions.

To reveal cytotoxicity induced by RNA-binding proteins containing PrLDs, we generated synthetic cDNAs encoding 67 repeats of the G/S-Y-G/S motif, 50 repeats of G/S-Y-G/S + Q or G/S-Y-G/S + Q/N motifs containing the PABP RRM, signal sequences for nuclear import/export, and GFP tags (Fig. 1a and Supplementary information). We assembled the base pairs necessary to encode the G**/**S-Y-G**/**S motif and minimize repeats of the nucleotide sequence to avoid formation of aberrant RNA structures. Additionally, we used the well-characterized PABP RRM from *Citrus sinensis*, which shares 43% identity with the human PABPN1^16^, to minimize the possibility of functional redundancy in mammalian cells. Furthermore, we added a NLS (MPKKKRKVGG) or NES (MLPPLERLTLDGG) to investigate compartment-specific cytotoxicity.

We inserted these cDNAs into a mammalian expression vector, followed by transfection into various cell lines and analysis by western blot. As shown in Fig. 1b, we detected full-length forms of the SYGQ-NES [predicted size: ~68 kDa; estimated isoelectric point (pI): 5.76; http://web.expasy.org/compute_pi/] and SYG-NES proteins (predicted size: ~68 kDa; estimated pI: 5.76) at ~73 kDa according to western blot results. Both artificial proteins were also detected within the stacking gel, indicating that both were prone to aggregation and formation of high-molecular-weight complexes. A series of deletion experiments revealed that the SYG ± Q PrLD was essential for the formation of these high-molecular-weight complexes (Fig. 1c). However, we observed low levels of the SYGQ/N-NES protein (predicted size: ~68 kDa; estimated pI: 5.76) in 293 T cells, with nearly undetectable formation of high-molecular-weight complexes (Fig. 1d).

Furthermore, soluble and insoluble fractionation showed that the SYG and SYGQ-NES proteins were present in the RIPA-insoluble and urea-soluble fractions, indicating that both formed strongly insoluble aggregates similar to candidate proteins found in the brains of ALS/FTD patients (Fig. 1e). Therefore, we focused on the SYG and SYGQ-NES proteins in subsequent experiments.

### Characterization of IBs formed in SYGQ-NES-expressing cells

**Figure 2:**
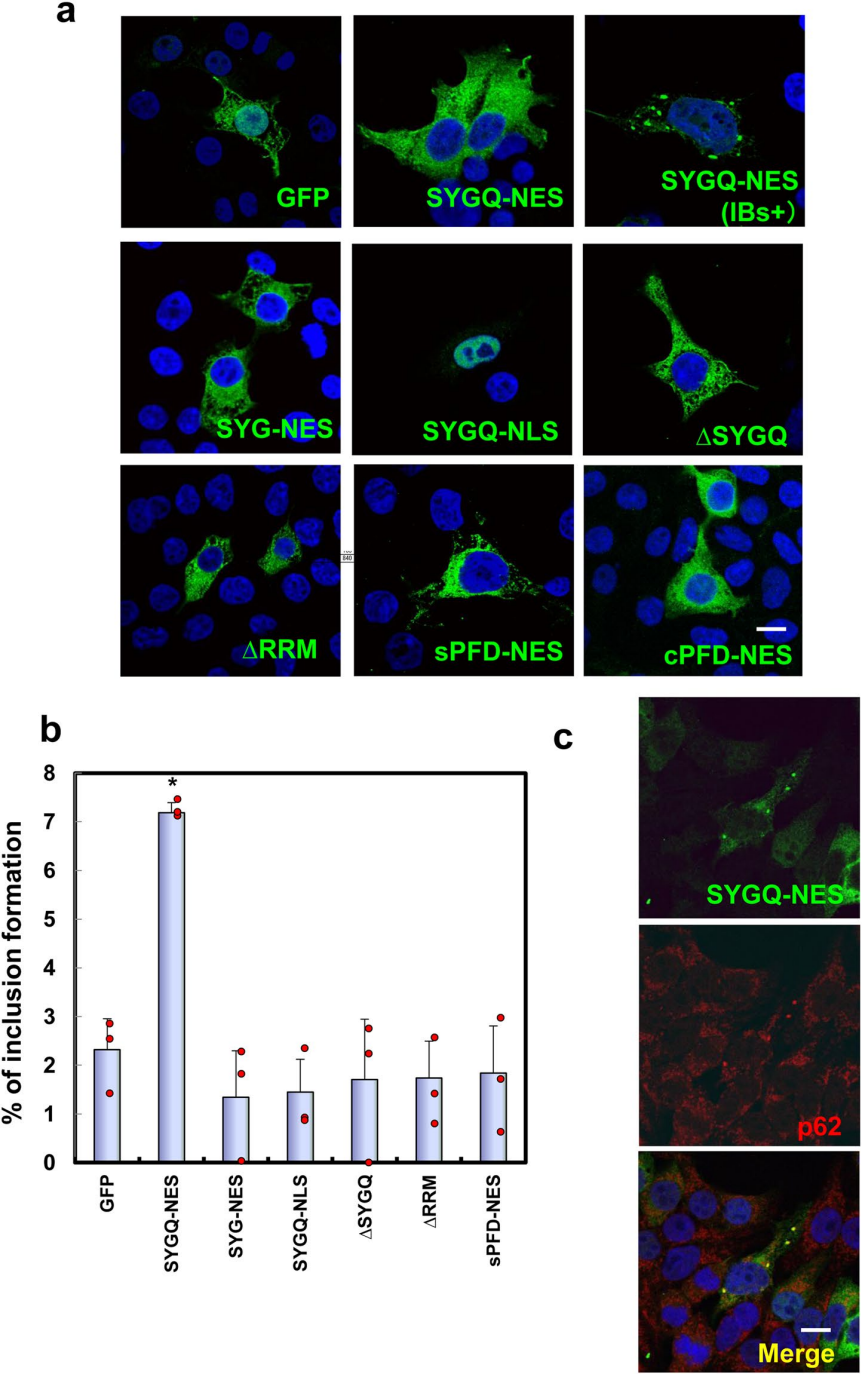
SYGQ-NES proteins form IBs in cultured cells. (**a**) At 48-h post-transfection, 293 T cells were counterstained with 4′,6-diamidino-2-phenylindole (DAPI) (blue). (**b**) Percentage of cells harboring IBs in ~300 transfected cells from three independent experiments (mean ± standard deviation). Plots indicate mean values from each experiment (n = 3). Scale bar: 10 μm. Asterisks (*) indicate significant differences between SYGQ-NES-GFP vs. GFP (control). *P < 0.0001, vs. GFP (One-way ANOVA). (c) Cytoplasmic IBs of SYGQ-NES are p62-positive. Scale bars: 10 μm.

A characteristic feature of ALS/FTD is the presence of IBs consisting of ALS-related RNA-binding proteins and immunoreactive to p62/sequestosome 1 throughout the affected regions. To examine subcellular localization of the artificial proteins, we performed immunocytochemistry in 293 T cells (Fig. 2a). Our results indicated that the SYGQ-NES protein formed characteristic p62-positive cytosolic IBs [mean percentage of cells with IBs ± standard deviation: 7.28 ± 0.28 (*n* = 3); Fig. 2b and c], but that this activity was rarely observed for the SYG-NES protein. Similar SYGQ-NES IBs were observed in other cell lines, including Neuro2a [percentage of cells with IBs: 10.1% (7 cells with IBs/69 cells expressing SYGQ-NES; *n* = 1)] and HeLa cells [percentage of cells with IBs: 3.8% (3 cells with IBs/79 cells expressing SYGQ-NES; *n* = 1)] (Supplementary Fig. 1).

IBs formed by molecules resulting from deletion experiments were rarely observed (Fig. 2 and Supplementary Fig. 2). Additionally, we also generated other artificial Q/N-rich prion-like proteins, synthetic prion-forming domains (sPFD) designed by a prion-aggregation-prediction algorithm and control prion-forming domains (cPFD), which exhibit low prion propensity and used sPFD as a control (Supplementary information)^17^. Unexpectedly, western blot showed that cPFD, and not sPFD, formed high-molecular-weight complexes in the stacking-gel for unknown reasons (Supplementary Fig. 2). All mutant constructs, including those encoding the sPFD protein, showed rare IB formation (Fig. 2), indicating that the SYGQ-repeat PrLD, RRM, and cytoplasmic localization domains were critical for IB formation.

**Figure 3:**
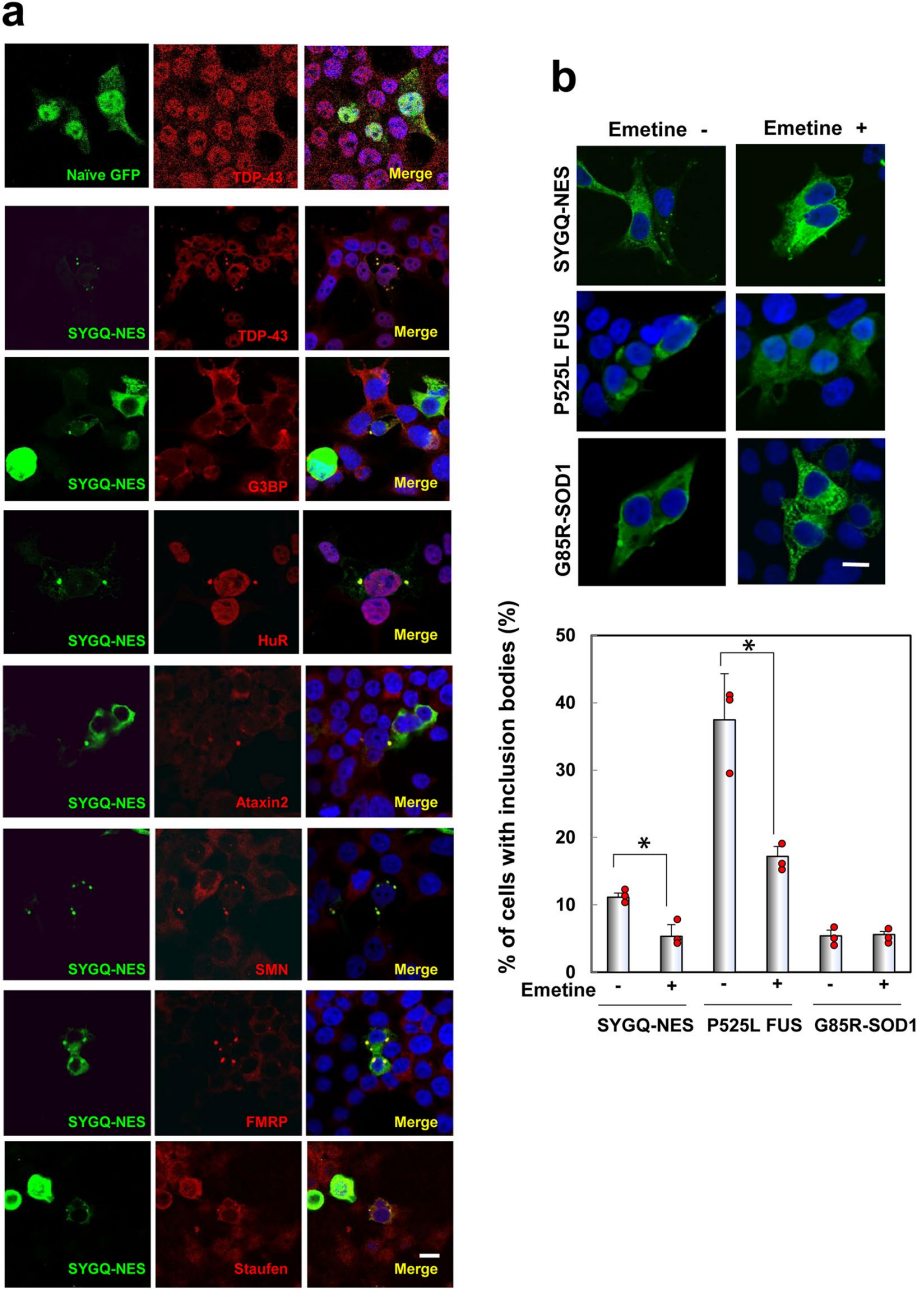
Interactions between SYGQ-NES and other ALS-related RNA-binding proteins in IBs. (**a**) 293 T cells expressing SYGQ-NES (green) were labeled with antibodies against TDP-43, G3BP, HuR, Ataxin2, SMN, FMRP and Staufen (red). Note that endogenous TDP-43 is predominantly nuclear but partially cytoplasmic in 293 T cells expressing naïve GFP (green) as control. Scale bar: 10 μm. (**b**) Emetine disassembles SYGQ-NES IBs. Treatment with 10 μg/mL emetine for 1 h resulted in disassembly of cytoplasmic IB formation in 293 T cells expressing SYGQ-NES-GFP and mutant FUS-GFP, but not those expressing mutant SOD1-GFP. Plots indicate mean values from each experiment (n = 3). Scale bar: 10 μm. *P < 0.05, vs. no treatment (Student’s *t* test). Plots indicate mean values of each experiment.

Previous studies demonstrated that IBs involving TDP-43 and FUS in ALS/FTD-patient brain tissue recruit other RNA-binding proteins, such as components of SGs^8,10,13,18–20^. As shown in Fig. 3a, SYGQ-NES IBs exhibited co-localization with endogenous TDP-43 [percentage of TDP-43^+^ IBs: 58.5% (24 TDP-43^+^ IBs/41 IBs; *n* = 1)], SG markers {[Ras GTPase-activating protein-binding protein 1 (G3BP); percentage of G3BP^+^ IBs: 32.4% (12 G3BP^+^ IBs/37 IBs; *n* = 1)], HuR [percentage of HuR^+^ IBs: 12.5% (5 HuR^+^ IBs/40 IBs; *n* = 1)], Ataxin2 [percentage of Ataxin2^+^ IBs: 74.4% (29 Ataxin2^+^ IBs/39 IBs; *n* = 1)], and SMN [percentage of SMN^+^ IBs: 15.4% (6 SMN^+^ IBs/39 IBs; *n* = 1)]}, mRNA transporters, fragile X mental retardation protein (FMRP) [percentage of FMRP^+^ IBs: 52.5% (21 FMRP^+^ IBs/40 IBs; *n* = 1)], and Staufen [percentage of Staufen^+^ IBs: 26.8% (11 Staufen^+^ IBs/41 IBs; *n* = 1)]. Notably, SYGQ-NES IBs recruit components of SGs, including nuclear proteins such as TDP-43 and HuR; however, they show varying staining patterns, indicating that SYGQ-NES IBs possess partial features of SGs, and possibly belong to diverse subcellular entities known as heterogeneous RNA granules^6^.

Furthermore, administration of the inhibitor of translational elongation, emetine, stabilizes polysomes and suppresses SG formation^21^. As shown in Fig. 3b, treatment with this drug dispersed IBs [(mean percentage of cells containing IBs formed under normal conditions in the presence and absence of emetine: 11.1 ± 0.6% vs. 5.3 ± 1.7%, respectively (*P* < 0.05)] in 293 T cells expressing SYGQ-NES and also mutant FUS^10^, but not aggresomes containing mutant forms of superoxide dismutase 1. These findings strongly suggested that SYGQ-NES IBs exhibited characteristics associated with SGs.

**Figure 4:**
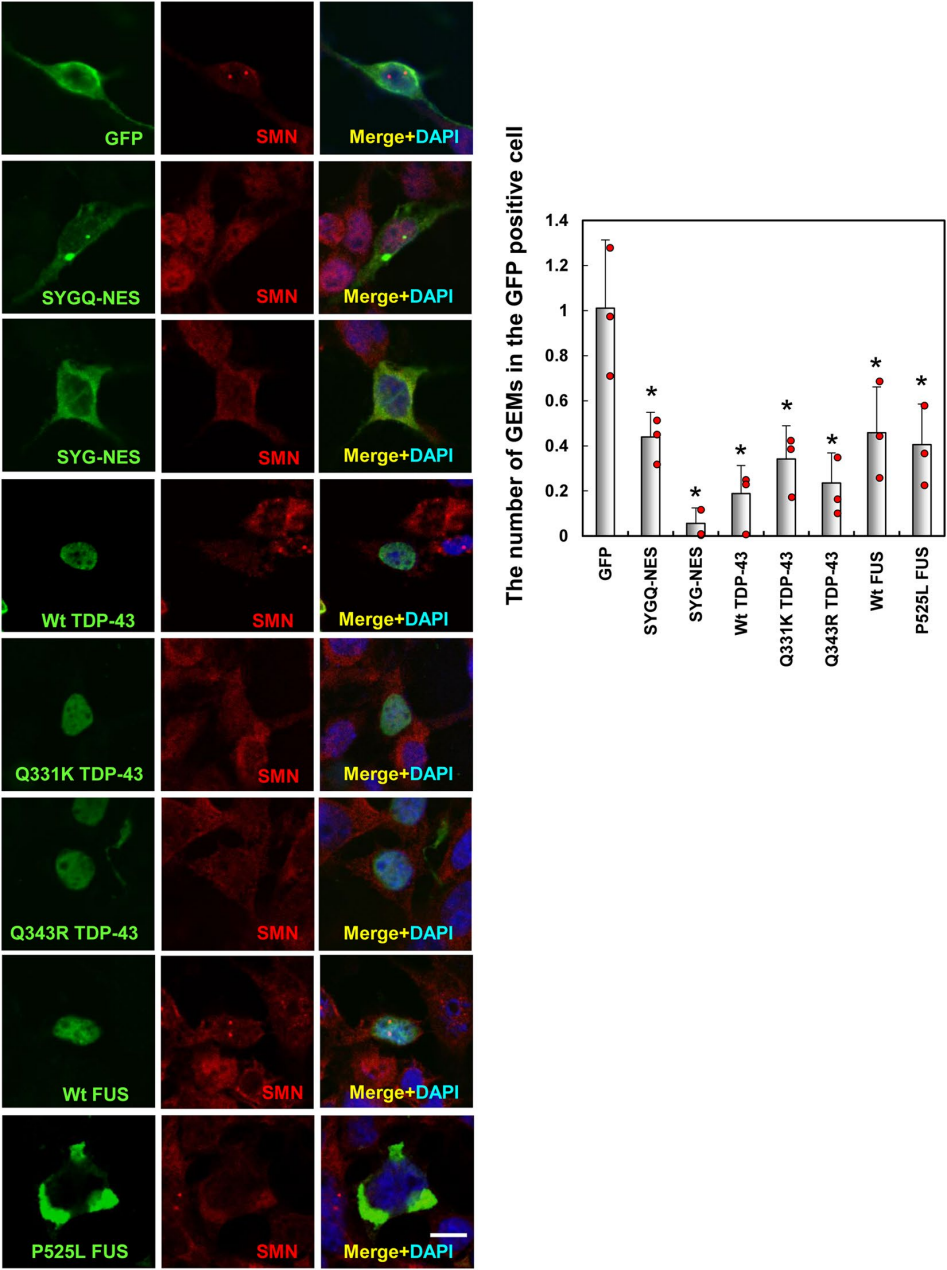
GEM number following expression of artificial RNA-binding proteins containing PrLDs. HeLa cells, in which GEM is well-observed, transiently expressed SYG or SYGQ-NES (green). Numbers of GEM formation was assessed by immunostaining with anti-SMN (red). Graphs show the average number of GEMs formed in cells expressing SYG or SYGQ-NES (*n* = 3). *P < 0.002, vs. GFP (One-way ANOVA). Scale bar: 10 μm

Furthermore, ALS-causative mutations in FUS abnormally interact with SMN and lead to loss of nuclear Gemini of coiled bodies (GEM), and mutation of TDP-43 or FUS disrupt GEM assembly or stabilization^22–26^. Here, we observed that expression of SYG/SYGQ-NES proteins significantly reduced nuclear GEMs consistent with results observed in the presence of other ALS-linked RNA-binding protein (Fig. 4).

### SYG/SYGQ-NES proteins are recruited to SGs

**Figure 5:**
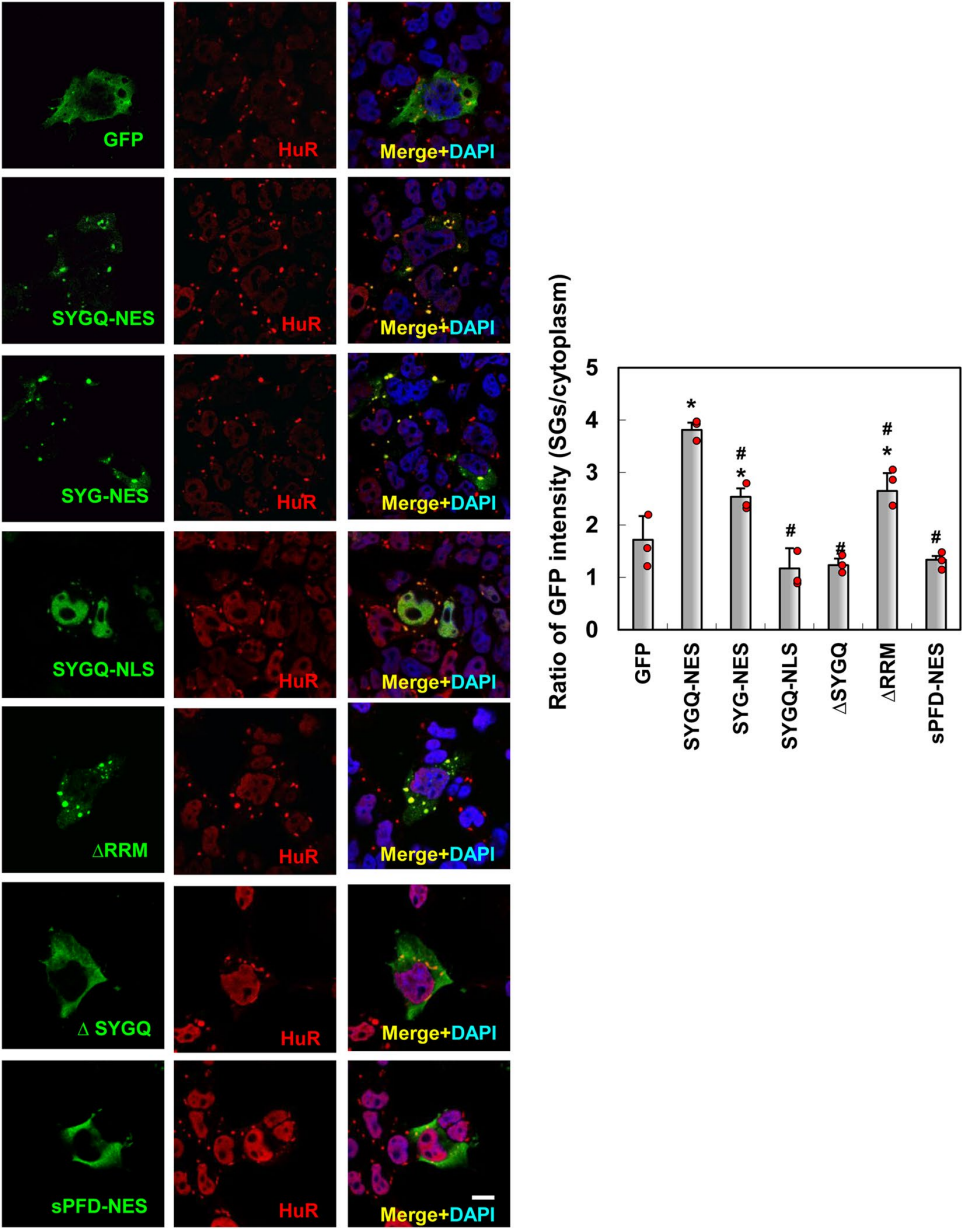
SYG/SYGQ-NES is recruited to SGs in arsenite-treated HeLa cells. Following 1 h treatment with 0.5 μM arsenite, HeLa cells were fixed and immunostained for SG markers and with the anti-HuR antibody. Graphs indicate the ratio of the mean intensity of GFP in SGs to that observed in the cytoplasm. The fluorescent distributions in SGs from cells expressing SYG/SYGQ-NES and ΔRRM were higher than those observed in cells expressing GFP alone. *P < 0.003, vs. GFP; ^#^*P* < 0.0002; vs. SYGQ-NES (one-way ANOVA). Scale bar: 10 μm.

**Figure 6:**
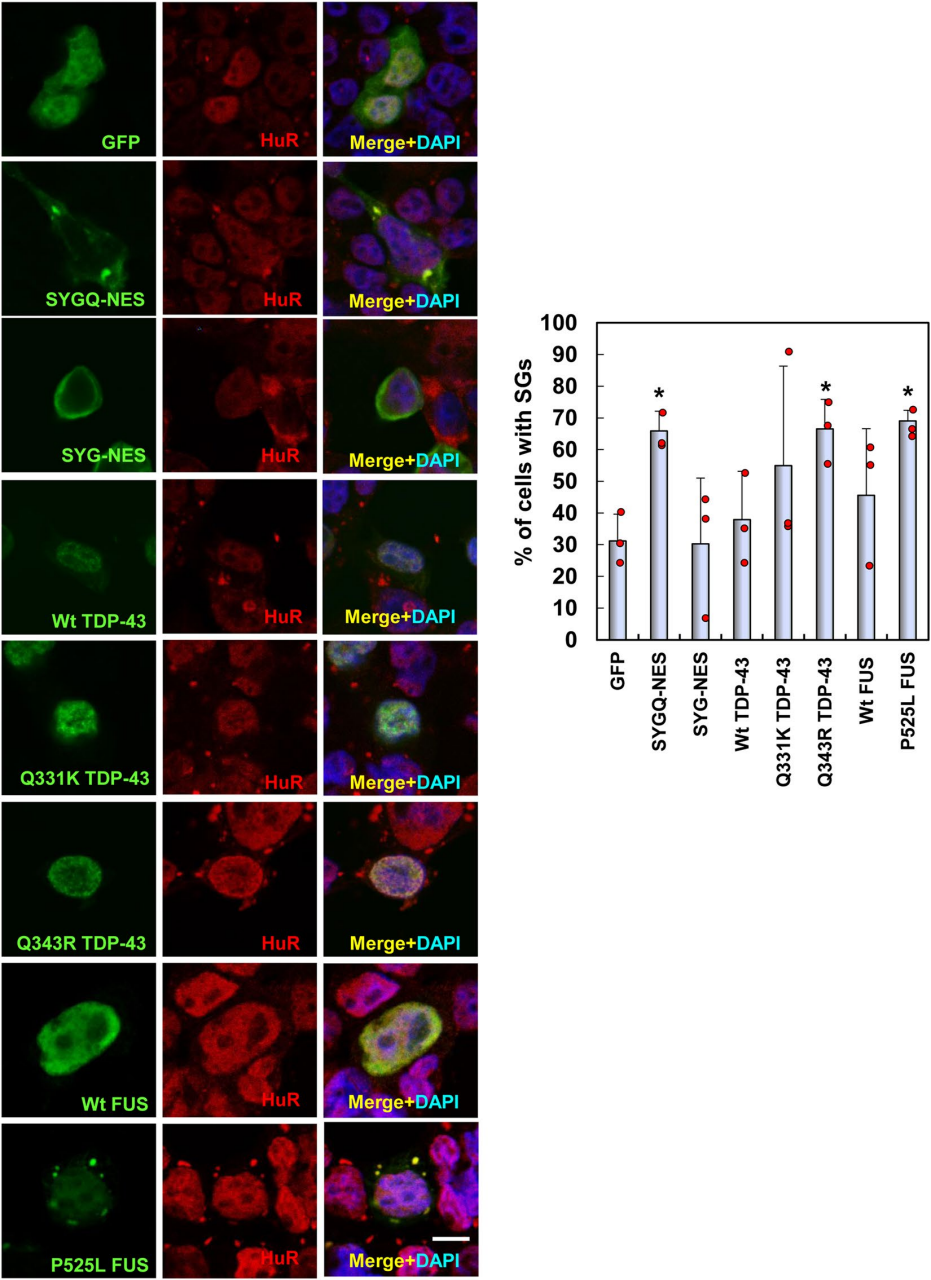
SG formation involving artificial RNA-binding proteins containing PrLDs. HeLa cells expressing GFP, SYG/SYGQ-NES, TDP-43-GFP, or FUS-GFP were double labeled with monoclonal anti-GFP and -HuR antibodies following a 1 h treatment with arsenite (0.5 mM). Approximately 25 transfected cells from three independent experiments were used to quantify the number of SGs formed per cell. *Indicates statistically significant differences (*P < 0.04 vs. GFP, One-way ANOVA). Scale bar: 10 μm.

SGs are formed as cytoplasmic foci that aggregate mRNA and RNA-binding proteins under stress conditions, such as hypoxia and heat shock. SGs function as part of the RNA quality control system by stabilizing and storing mRNAs and arresting translation to prevent the accumulation of misfolded proteins^27^. ALS-linked RNA-binding proteins TDP-43, FUS, and Ataxin2 are SG components, and their mutation leads to the formation of aberrant SGs *in vitro* and *in vivo*^8–11,18^. To examine whether SYGQ-NES proteins are recruited to SGs, we performed double immunofluorescence staining in HeLa cells, which is commonly used for examining the characters of SG^6–8^ after treatment with arsenite, a well-established SG inducer (38). As shown in Fig. 5, both artificial proteins, predominantly SYGQ-NES, were recruited to SGs following arsenite treatment. A series of deletion experiments revealed that SYG/SYGQ repeats, cytoplasmic localization domains, and partial Q-rich domains and RRMs were critical for SG localization. Furthermore, analysis of the number of HeLa cells expressing SYG/SYGQ-NES proteins with arsenite-induced SGs showed that SYGQ-NES expression increased SG formation in a manner similar to that observed with the ALS mutants TDP-43 (Q343R) and FUS (P525L) (Fig. 6). These findings indicated that SYGQ-NES expression dysregulated SG formation similar to results observed in the presence of ALS-associated mutants^8–11^.

### SYG/SYGQ-NES cytotoxicity

**Figure 7:**
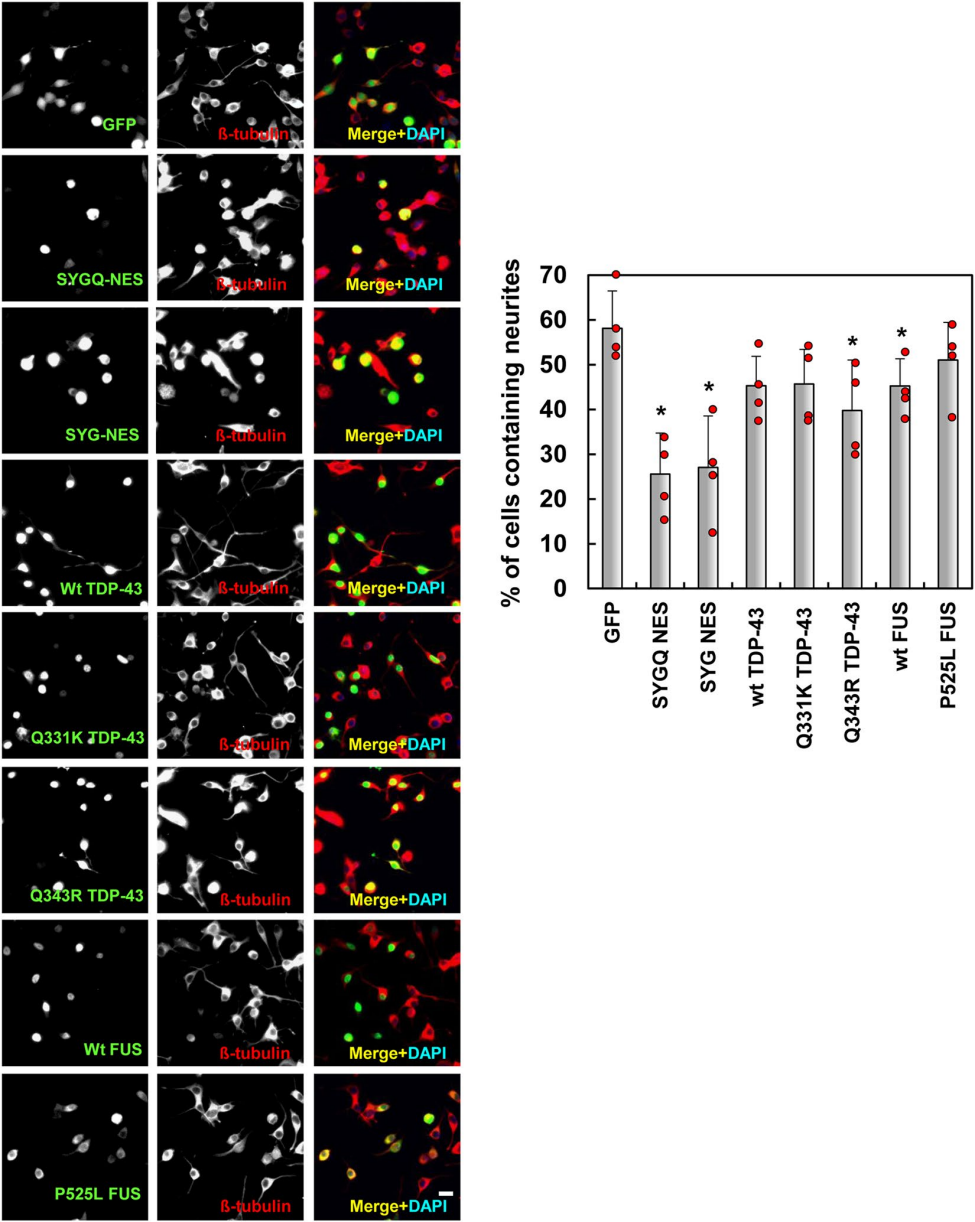
Neurites in differentiated Neuro2a cells expressing SYG/SYGQ-NES. Representative images of immunocytochemistry for SYG/SYGQ-NES-positive differentaited Neuro2a cells using the anti-βIII-tubulin antibody. Graphs indicate quantitative data from neurite-bearing cells. Neuro2A cells containing neurites exhibiting diameters of at least two cell-bodies in length were scored as neurite-bearing cells (*n* = 3 independent experiments; mean ± standard deviation). *P < 0.05, one-way ANOVA. Scale bar: 20 μm.

**Figure 8:**
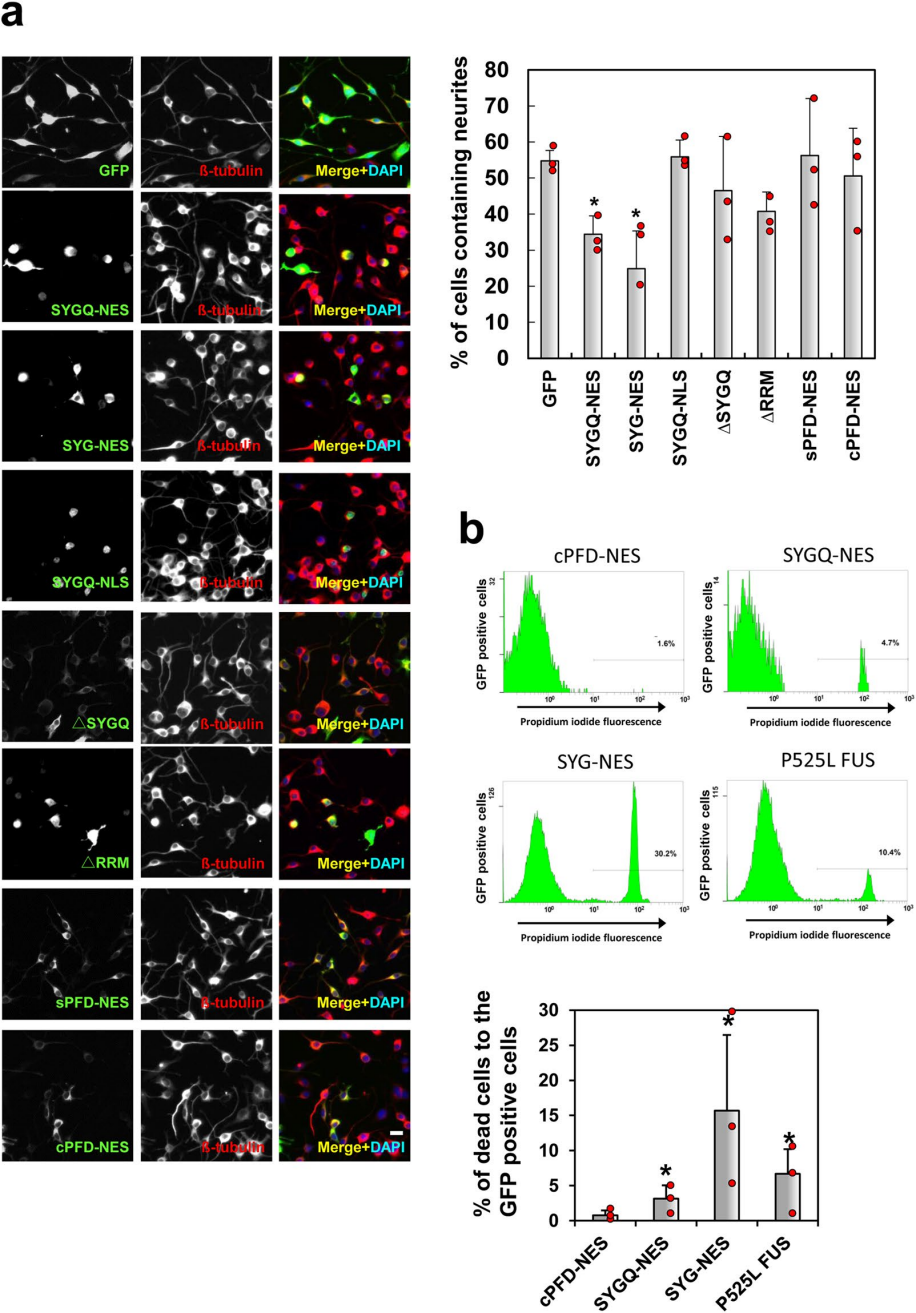
Cytotoxicity induced by SYG/SYGQ-NES. (**a**) Neurites in differentiated Neuro2a cells expressing deletion variants of SYG and SYGQ-NES. Representative images of βIII-tubulin immunostaining of differentiated Neuro2a cells. sPFD-NES and cPFD-NES were used as a negative control. Graphs indicate quantitative data of βIII-tubulin-positive neurite-bearing neurons (*n* = 3 independent experiments; mean ± standard deviation) *P < 0.03 (One-way ANOVA). Scale bar: 20 μm. (**b**) Dead cells to the GFP positive cells by fluorescence-cell sorting. Neuro2a cells were subjected to transfection with SYG, SYGQ-NES, or mutant FUS tagged with GFP, followed by PI staining after a 48 h incubation for the detection of dead (GFP+PI+) cells. cPFD-NES, which shows similar GFP intensity as SYG and SYGQ-NES, was used as a negative control. Representative fluorescence-activated cell sorting pictograms. The percentage of dead (GFP+PI+) cells are shown, and the graph indicates the mean ± standard deviation (*n* = 5). Asterisks *indicate a significant differences (*P < 0.05; Student’s t test).

To determine whether SYG/SYGQ-NES expression leads to neurotoxicity, we measured neurite length on differentiated Neuro2a cells, a well-established model of neurite outgrowth^28–30^. SYG/SYGQ-NES-expressing cells become rounded and shrink, and showed shorter neurites compared with those observed on control cells (Fig. 7). We also observed shorter neurites in cells containing ALS-related TDP-43 (Q343R) and wild type FUS constructs. Additionally, deletion experiments revealed that SYG/SYGQ repeats, cytoplasmic localization domains and RRMs were critical for the disturbance of neurite outgrowth (Fig. 8a). We then assessed the induction of cell death following transfection of the artificial molecules as determined by propidium iodide-positive (PI+) cells in GFP-positive Neuro2a cells and cell sorting. As shown in Fig. 8b, we observed increased levels of PI + cells expressing both artificial proteins, but higher levels in the presence of SYG-NES, indicating that expression of either SYG or SYGQ-NES resulted in cytotoxicity.

## Discussion

Increasing evidence supports hypotheses involving gain of toxic functions resulting from cytoplasmic accumulation of RNA-binding proteins involved in ALS/FTD pathogenesis. Overexpression of human TDP-43 and FUS in mice causes progressive motor-neuron degeneration similar to ALS phenotypes, including cytoplasmic aggregation^31–35^. However, several FUS-knockout mutant models do not exhibit phenotypes associated with motor-neuron dysfunction^36,37^, although expression of mutant FUS can also directly recapitulate ALS-like motor-neuron abnormalities in mice independent of reductions in FUS activity^38,39^. Haploinsufficiency mutations involving FUS and that lead to nonsense-mutation-mediated mRNA decay were identified in an essential-tremor-affected family that did not reveal any signs of ALS^40^. Therefore, these findings suggested that mislocated RNA-binding proteins are critical for neurodegeneration development in a gain of toxic function manner.

Our findings provided several insights into the cellular-biochemical features of ALS/FTD-linked RNA-binding proteins. Artificial RNA-binding proteins containing SYG/SYGQ repeats initiated aggregation and assembly with SG components and caused neuronal toxicity. Low-complexity sequences referred to as G/S-Y-G/S motifs, cytoplasmic localization and RRMs are essential for inhibition of neurite outgrowth (Fig. 8). SG localization requires G/S-Y-G/S motifs, cytoplasmic localization domains, partial Q-rich domains, and RRMs (Fig. 5), and IB formation also requires Q-enriched G/S-Y-G/S motifs, cytoplasmic localization domains, and RRMs (Fig. 2). Notably, the SYG-NES protein showed formation of high-molecular-weight complexes according to western blot analysis (Fig. 1), but we rarely observed IB formation in cells expressing SYG-NES proteins (Fig. 2). Although it might be possible that cells expressing SYG-NES proteins were unhealthy (rounded, shrunken, and floating) prior to the maturation of visible IBs, IB formation might be required for additional cellular process(es) beyond characteristics required formation of high-molecular-weight complexes. These characteristics were distinct from those observed in a protein harboring a *de novo* design of sPFDs^17^ (Figs 2,5, and 8) and other ALS-linked aggregated proteins, such as SOD-1 (Fig. 3b), indicating that the artificial SYG/SYGQ-NES proteins lacking specific physiological functions associated with target RNAs effectively mimicked specific pathological features associated with ALS/FTD-linked RNA-binding proteins.

Although the detail pathological mechanism of the SYG/SYGQ-NES protein remains unknown and requires further study, recent evidence suggests that ALS-linked RNA-binding proteins, such as TDP-43 and FUS, are mislocated to the cytoplasm and might affect target RNAs and other RNA-binding proteins, resulting in impairment of RNA metabolism and RNA quality control mechanisms, such as those associated with SGs, during neurodegeneration^1,41^. The SYG-NES protein is strongly cytotoxic in the absence of IBs, indicating that IB formation is unnecessary for cytotoxicity (Figs 2 and 8); however, high-molecular-weight complexes of low-complexity sequences, including G/S-Y-G/S motifs, might interfere with other RNA-binding proteins, including TDP-43 and other SG components, resulting in cell toxicity as a result of RNA dysregulation.

Several points should be kept in mind during the exploration of ALS/FTD pathogenesis using artificial SYG/SYGQ-NES proteins, and alternative interpretations should be considered. First, although most ALS/FTD-linked RNA-binding proteins are ubiquitously expressed in all tissues, ALS/FTD is a degenerative disease that predominantly affects the frontal lobe containing primary and secondary motor neurons from the spinal cord, which are anatomically related. Therefore, future studies need to confirm motor-dominant neurodegeneration following SYG/SYGQ-NES expression under the control of a ubiquitous promoter in transgenic animals.

Second, abundant molecular and cellular evidence suggests that the misfolding of specific proteins, such as α-synuclein, β-amyloid, and tau, can act as seeds for aggregation and can be transmitted intercellularly, thereby constituting a manner of self-dissemination similar to that observed with prions^42^. Recent neuropathological studies of postmortem brains from ALS patients described a pattern of phospho-TDP-43 immunoreactivity that agrees with a systematic spreading of TDP-43-related pathology^43,44^. Cell-biological studies also showed TDP-43 soma-to-soma transmission between cells and propagation within neuroanatomical systems across axon terminals^45^. Therefore, future studies should examine whether artificial proteins harboring low-complexity sequence(s) in PrLD(s) also exhibit similar mechanisms of seeded aggregation and propagation.

Third, affected regions in most ALS patients are characterized by TDP-43-positive IBs in the cytoplasm and also by the depletion of TDP-43 protein in the nucleus^46,47^. Analyses by cross-linking and immunoprecipitation suggested that there might be thousands of RNA molecules targeting TDP-43 and FUS during the mRNA-maturation and gene-regulation processes^48–50^. A study using a conditional mouse TDP-43 gene-targeting approach in spinal cord motor neurons reported progressive development of ALS-related phenotypes^51^. Therefore, theories supporting a loss-of-function phenotype associated with TDP-43 proteinopathy and leading directly to RNA dysregulation and development of neurodegeneration remain plausible^46,47,52^. In this study, we were unable to directly examine loss-of-function phenotypes associated with ALS pathogenesis using artificial proteins.

Forth, analysis by cross-linking and immunoprecipitation of sequences suggested that approximately 30% mRNA of the murine transcriptome interacted with TDP-43 and FUS^48–50^. Therefore, FUS and TDP 43-proteinopathies possibly cause gross RNA dysregulation rather than specific mRNA disruptions in ALS/FTD development. Therefore, an RRM of PABP from *Citrus sinensis*, which represents an authentic ortholog of the animal PABPs, was used for these artificial proteins to avoid specific binding to RNA in mammalian cells. We speculated that the overexpressed plant-based RRM interacted widely and non-specifically with mRNAs and caused gross RNA dysregulation; however, this does not exclude the possibility that off-target tight-binding to unexpected RNAs might affect the interpretations in studies using *de novo* proteins. Future studies should define comprehensive artificial SYG/SYGQ-NES proteins -RNA interaction and compare it with other ALS-linked RNA binding protein interactions.

Finally, most neurodegeneration-associated aggregated proteins, such as tau and TDP-43, are easily targeted by protein degradation systems and are multiply fragmented in the affected brain^47,53,54^. As shown in Fig. 1, many low molecular weight degradation products of SYG/SYGQ-NES were also detected using immunoblot analysis. Despite the presence of a prominent ~27 kDa fragment, which is predicted to be naïve GFP from the molecular weight, we cannot exclude the possibility of degradation products having unexpected effects and resulting in modified phenotypes. Here, the instability index for SYGQ-NES calculated by ProtParam (http://web.expasy.org/protparam/) was high (49.22), indicating that this must be improved to ensure stable expression in future studies. However, our findings suggested that the use of *de novo* proteins, specifically SYG/SYGQ-NES, effectively allowed the study of mechanisms associated with gain of toxic functions related to ALS/FTD pathogenesis. These results support the acquisition of an enhanced understanding of RNA dysregulation mediated by aggregated cytoplasmic RNA-binding proteins and promote the development of new strategies for the treatment of ALS/FTD proteinopathies.

## Materials and Methods

### cDNA and plasmids

cDNAs encoding SYG/SYGQ-NES/NLS and synthetic prion-forming domain (sPFD)/control prion-forming domain (cPFD)-NES/NLS (Sequences are shown in Supplementary information) were synthesized (GenScript, NJ, USA) and subcloned into pcDNA (3.1) or pEGFP-N1 (Clontech, CA, USA) or. Wild type and mutant TDP-43 and FUS were described in previous papers^8,10^.

### Cell culture

HeLa human carcinoma cells, human embryonic kidney 293 cells (293 T cells), were maintained in Dulbecco’s modified Eagle’s medium (Gibco, Grand Island, NY) containing 10% fetal bovine serum. Neuro2a neuroblastoma cells were cultured in αMEM (Gibco) supplemented with 10% fetal bovine serum. Transfections were performed using Lipofectamine 2000 (Life Technologies) according to the manufacturer’s instructions. Neuro2a cells were differentiated where stated by incubating in DMEM supplemented with 2% (vol/vol) FBS and 20 μM retinoic acid (Sigma-Aldrich, St. Louis, MO, USA) for 24 h.

### Antibodies and reagents

Mouse monoclonal anti-ubiquitin, HuR, and GFP were purchased from Santa Cruz Biotechnology (Santa Cruz, CA, USA). Mouse monoclonal anti-α tubulin was from Invitrogen. Mouse monoclonal Anti-βIII Tubulin (2G10), p62/SQSTM1, MAP2 and FMRP was obtained from Millipore (Billerica, MA). Rabbit polyclonal anti-N-terminus FUS was from Bethyl Labs (Montgomery, TX, USA). Anti-TDP-43 and G3BP antibodies were from Proteintech (Chicago, IL, USA). Rabbit polyclonal anti-GFP antibody was from Rockland Immunochemicals Inc. (Gilbertsville, PA, USA). Mouse monoclonal anti-GFP antibody was from Santa Cruz Biotechnology, Inc (Dallas, TX, USA).Mouse Anti-Ataxin-2 was purchased from BD Biosciences (Sparks, MD, USA). Rabbit polyclonal anti-GAPDH antibodies were from Cell Signaling Technology (Beverly, MA, USA). Propidium iodide was purchased from Dojindo (Kumamoto, Japan). Emetine, and Arsenite was purchased from Sigma-Aldrich.

### Immunoblot analysis

Cells were briefly sonicated in cold lysis buffer [50 mM Tris-HCl, pH 7.4, 150 mM NaCl, 0.5% NP-40, 0.5% sodium deoxycholate, 0.25% sodium dodecyl sulfate, 5 mM EDTA, and protease inhibitor cocktail (Sigma-Aldrich)]. Cell lysates were separated using SDS-PAGE with 10% Tris-glycine gel, after which proteins were transferred to a polyvinylidene difluoride membrane (Millipore, Billerica, MA). The membrane was incubated with primary antibodies, followed by horseradish peroxidase-conjugated secondary antibodies and then visualized using enhanced chemiluminescence reagents (PerkinElmer Life Sciences, Boston, MA, USA). Protein levels were determined by densitometry using an Epson ES-2000 scanner and Image J (National Institutes of Health, Bethesda, MD, USA).

To examine the solubility profile of artificial protein, sequential extractions were performed. The cells were lysed in cold RIPA buffer (50 mM Tris, 150 mM NaCl, 1% NP-40,5 mM EDTA, 0.5% sodium deoxycholate, and 0.1% SDS, pH 8.0 + Protease inhibitors). The cell lysates were cleared by centrifugation at 100,000 × g for 30 min at 4 °C to generate the RIPA soluble samples. To prevent carry-overs, the resulting pellets were washed with RIPA buffer twice. RIPA insoluble pellets were then extracted with urea buffer (7 M urea, 2 M thiourea, 4% CHAPS, 30 mM Tris, pH 8.5 + Protease inhibitors), and centrifuged at 100,000 × g for 30 min at 22 °C.

### Immunofluorescence with cultured cells

At 48 h after transfection, cells were fixed with 4% paraformaldehyde on ice for 10 min and then permeabilized in 0.2% Triton X-100 at room temperature for 10 min. Post-fixation with 4% paraformaldehyde on ice for 5 min followed. After blocking of nonspecific binding, coverslips were incubated with primary antibodies and then incubated with Alexa 488 and/or Alexa 586-conjugated secondary antibodies (Invitrogen). Immunofluorescent staining was examined using a Leica TCS SP5 confocal microscope (Leica, Wetzlar, Germany).

Well-defined cytoplasmic foci of 1.0–2.5 μm diameter were counted as IBs. To detect cell death, cell were trypsinized and stained with 1 μg/ml propidium iodide (Dojindo, Japan). Cell sorting was performed using MoFlo XDP (Beckman Coulter, IN, USA).

For cytotoxicity analysis in differentiated Neuro2a cells, neurites in more than 30 transfected cells from independent experiments (n = 3) were evaluated using ImageJ, and cells containing neurites of at least two cell-body-diameter length were scored as neurite-bearing cells^28,29^.

### Statistical analysis

Statistical analysis of the data was performed by one-way ANOVA with Fisher’s protected least squares difference test or Student’s t-test using the Statview 5.0 system (Statview, Berkley, CA, USA).

## Electronic supplementary material

Supplementary Information

Supplementary Information
