# Author Correction: *De novo* design of RNA-binding proteins with a prion-like domain related to ALS/FTD proteinopathies

**DOI:** 10.1038/s41598-020-65024-x

**Published:** 2020-05-26

**Authors:** Kana Mitsuhashi, Daisuke Ito, Kyoko Mashima, Munenori Oyama, Shinichi Takahashi, Norihiro Suzuki

**Affiliations:** Departments of Neurology, Keio University School of Medicine, 35 Shinanomachi, Shinjuku-ku, Tokyo 160-8582 Japan

Correction to: *Scientific Reports* 10.1038/s41598-017-17209-0, published online 04 December 2017

A supplementary file containing the sequence information was omitted from the original version of this Article. This has been corrected in the HTML version of the Article; the PDF version was correct at time of publication.

