# Supplementary material for: De novo design of RNA-binding proteins with a prion-like domain related to ALS/FTD proteinopathies
Source: Sci Rep. 2017 Dec 4;7:16871. doi: 10.1038/s41598-017-17209-0 (PMC5715010; doi:10.1038/s41598-017-17209-0)

De novo design of RNA-binding proteins with a prion-like domain related to ALS/FTD proteinopathies.

Kana Mitsuhashi, Daisuke Ito, Kyoko Mashima, Munenori Oyama, Shinichi Takahashi and Norihiro Suzuki

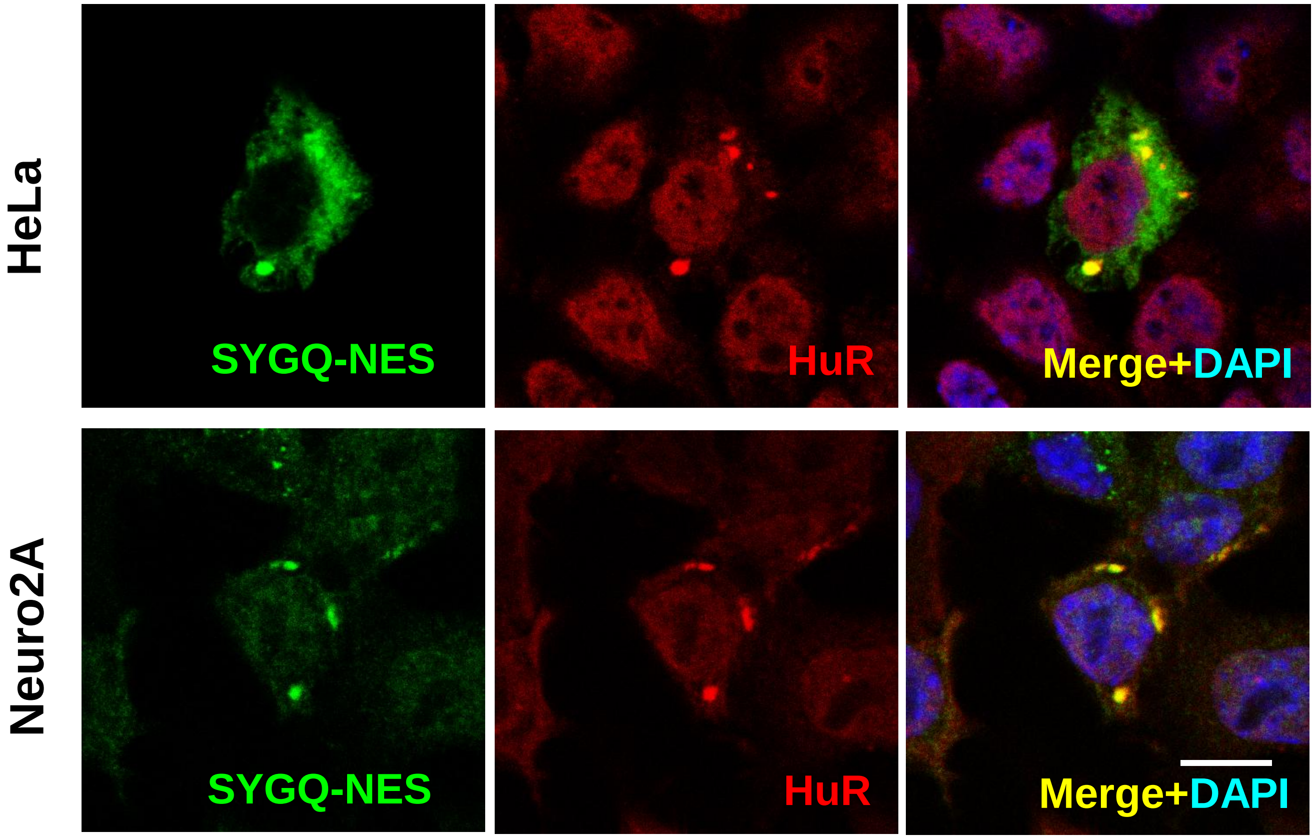

Supplementary Figure 1. SYGQ-NES proteins form IBs in cultured cells. At 48-h post-transfection, HeLa and Neuro2A cells expressing SYGQ-NES (green) were labeled with antibodies against SG marker HuR (red) and counterstained with DAPI (blue). Scale bars: 10 μm.

De novo design of RNA-binding proteins with a prion-like domain related to ALS/FTD proteinopathies.

Kana Mitsuhashi, Daisuke Ito, Kyoko Mashima, Munenori Oyama, Shinichi Takahashi and Norihiro Suzuki

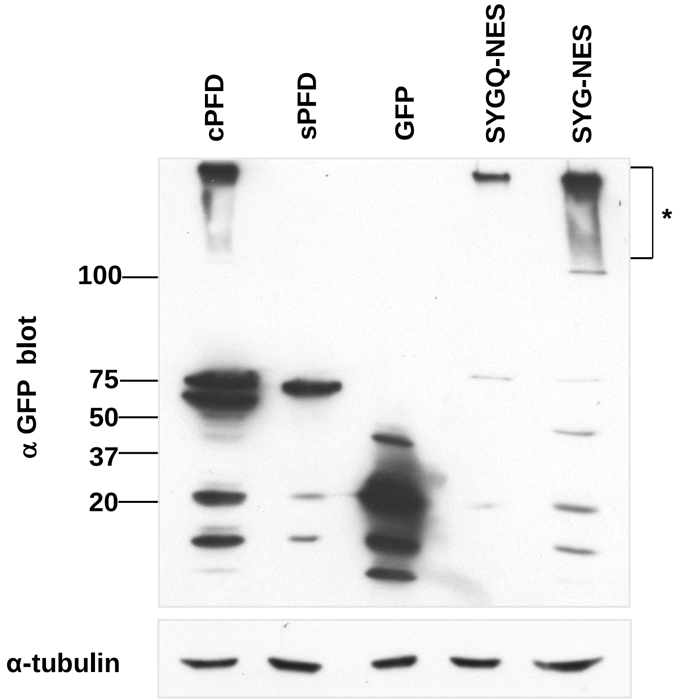

Supplementary Figure 2. Expression of artificial Q/N-rich prion-like proteins, sPFD and cPFD.

Immunoblot analysis of sPFD, cPFD, and SYG/SYG-NES constructs expressed in 293T cells. The proteins were loaded on a 10% Tris-glycine gradient gel and detected using an anti-GFP antibody for western blot analysis. Asterisks (*) indicate aggregation resolved using stacking gels.

De novo design of RNA-binding proteins with a prion-like domain related to ALS/FTD proteinopathies.
Kana Mitsuhashi, Daisuke Ito, Kyoko Mashima, Munenori Oyama, Shinichi Takahashi and Norihiro Suzuki

Non editing gel

Blue squares in the full-length blots are used for the cropped blots in Figure 1.

Figure 1 b

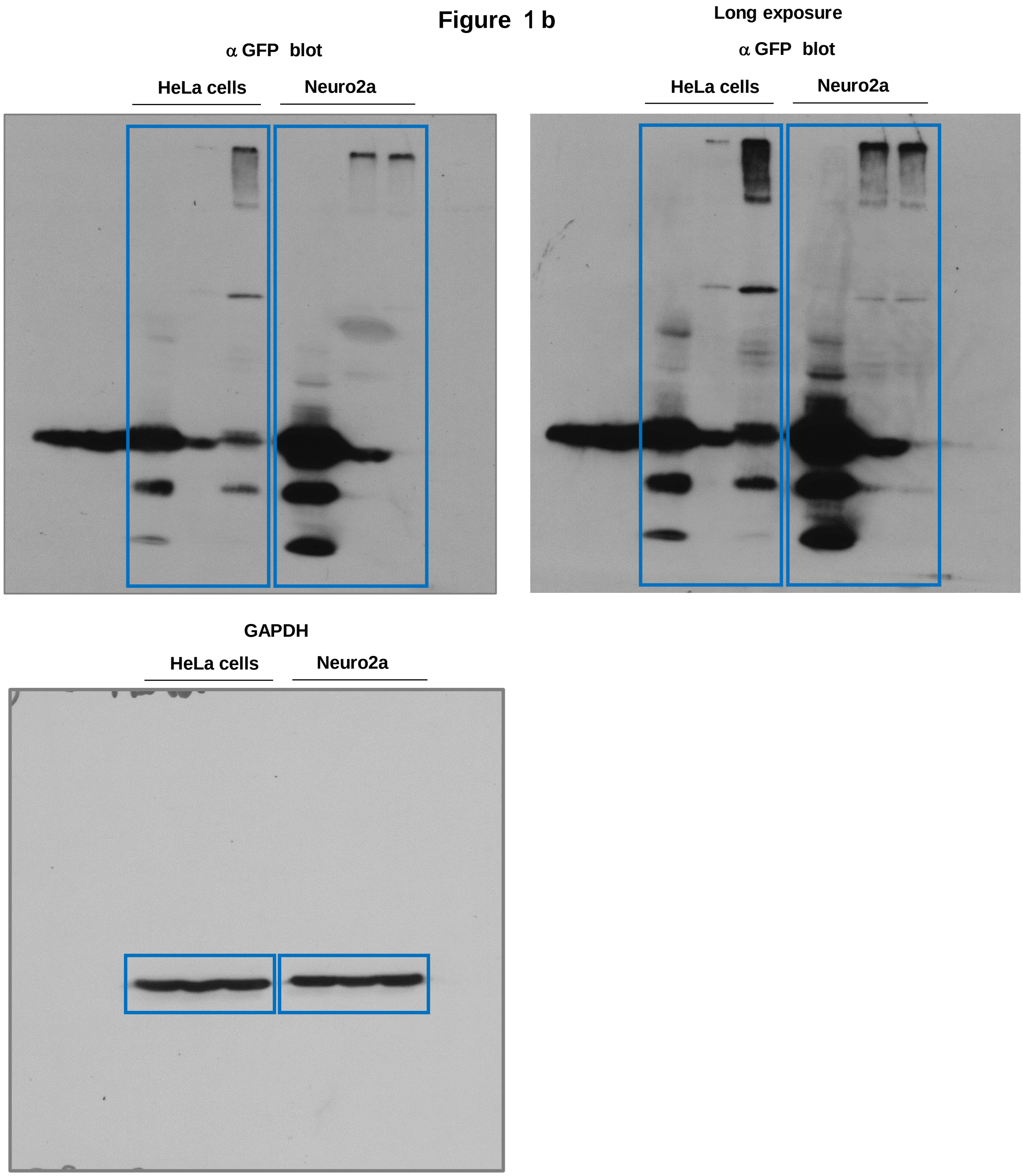

Non editing gel

Figure 1 c

α GFP blot | α-tubulin

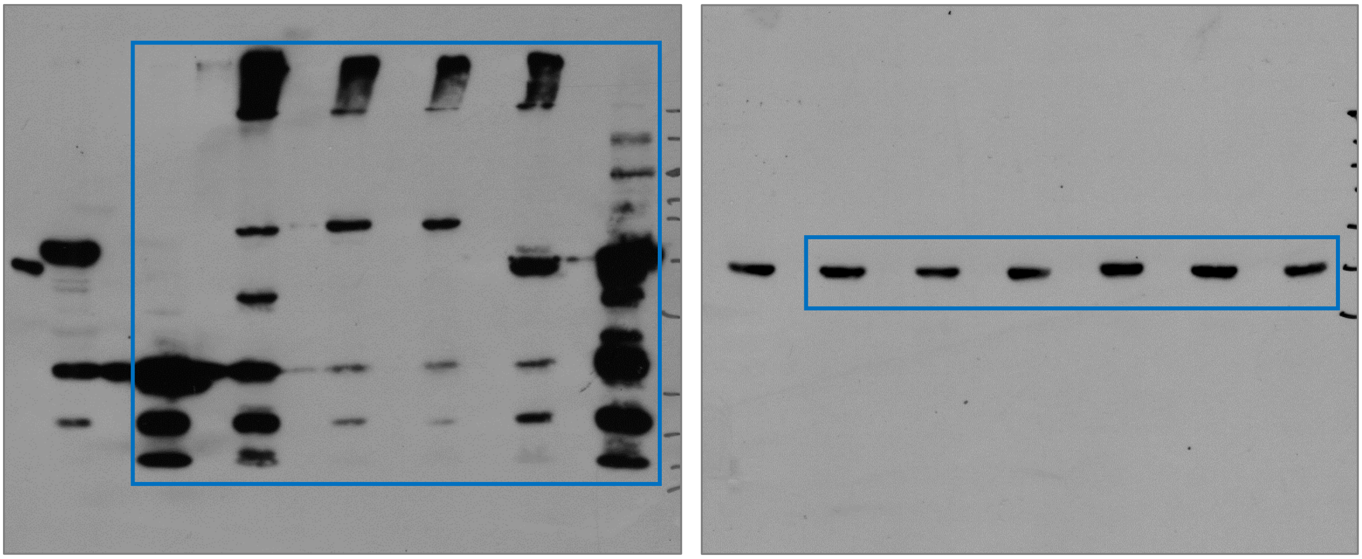

Figure 1 d

α GFP blot | α-tubulin

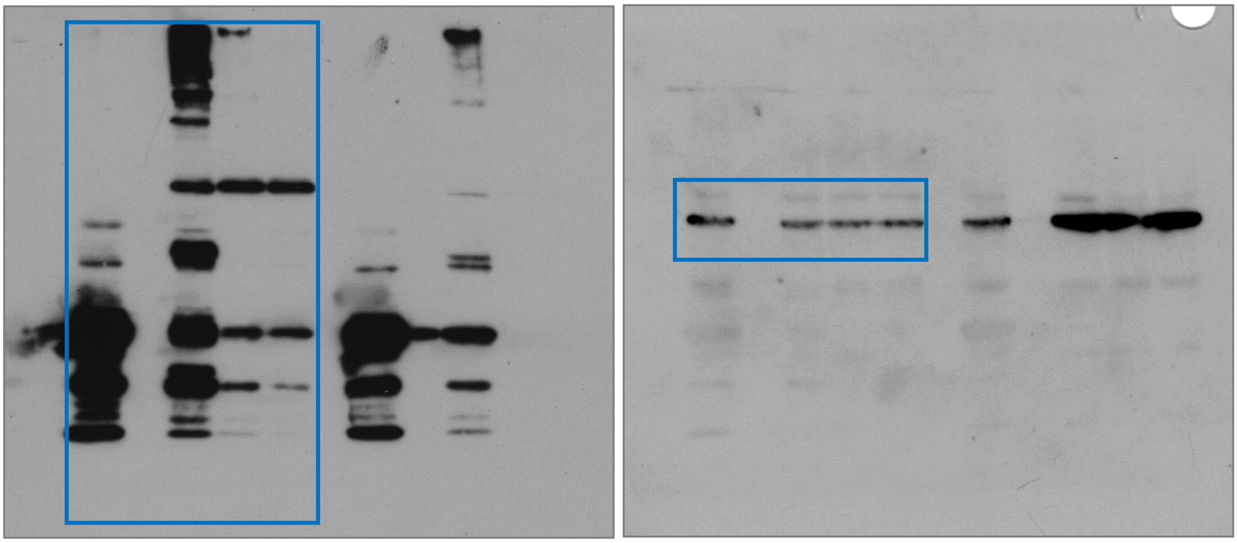

Non editing gel

Figure 1 e

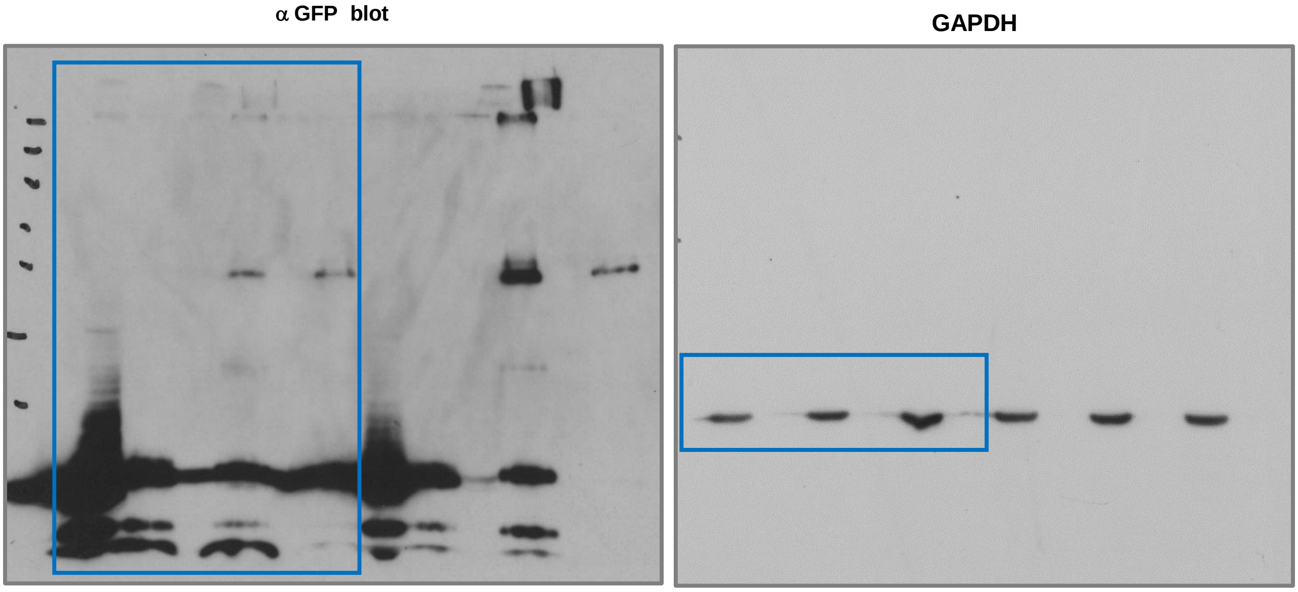

Non editing gel

Supplementary Fig. 2

α GFP blot

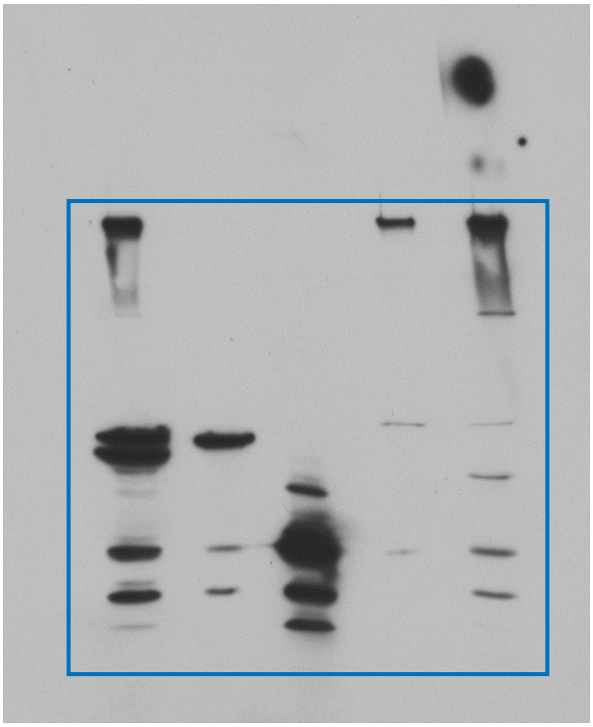

α-tubulin

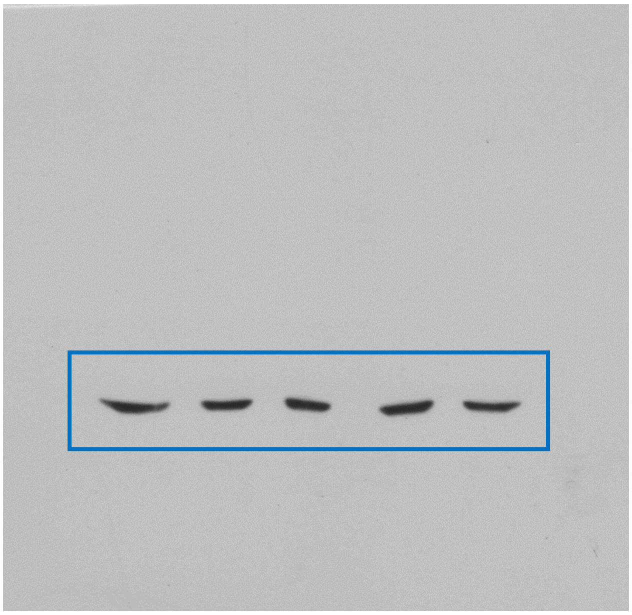

Supplement: Supplementary file 1 — Supplementary Information [file 41598_2017_17209_MOESM1_ESM.pdf]
