# Supplementary material for: De novo design of RNA-binding proteins with a prion-like domain related to ALS/FTD proteinopathies
Source: Sci Rep. 2017 Dec 4;7:16871. doi: 10.1038/s41598-017-17209-0 (PMC5715010; doi:10.1038/s41598-017-17209-0)
Supplement: Supplementary file 2 — Supplementary Information [file 41598_2017_17209_MOESM2_ESM.pdf]

De novo design of RNA-binding proteins with a prion-like domain related to ALS/FTD proteinopathies.

Kana Mitsuhashi, Daisuke Ito, Kyoko Mashima, Munenori Oyama, Shinichi Takahashi and Norihiro Suzuki'

SUPPLEMENTARY INFORMATION

SYG-NLS-NES-GFP cDNA

GCTAGCCTCGAGTCTAGAATGGGATCCTACTCCTCTTACGGCGGTTATGGAGGATATA

GTGGTTACAGTGGTTATGGGAGTTATGGGAGCTACAGCTCTTACGGAAGCTATTCCG

GTTACTCTGGATACGGCTCCTACTCATCCTACGGCGGGTACTCTGGTTACGGTGGCTA

CAGTGGGTATGGGGGATACGGCAGCTATGGATCTTATGGAAGCTACTCAGGATACGG

CGGTTATTCTTCATATTCTGGGTATGGTTCATATGGTGGGTATAGCGGATACTCCAGTT

ACGGTGGCTACGGATCCTATTCTTCATACAGTGGTTATTCAAGTTACTCAGGATATGG

CAGCTATTCTGGCTATGGAGGATACAGTAGCTATGGAGGATATTCTAGCTATGGAAGC

TACAGCGGCTATGGCAGCTATGGCAGCTATTCAGGATATGGAAGCTATTCCTCCTATTC

AGGCTATTCTGGGTATGGGTCTTATGGCGGATATTCTTCCTACTCTAGCTATGGTGGAT

ACGGGTCCTACGGAGGCTATGGTGGTTACTCAAGCTATAGTTCATATTCTGGATATAG

CTCTTATGGCGGTTATGGTGGCTATTCAGGTTACAGTGGATATGGTTCTAGAGAATTCT

CCAAGGATCTTGAGGACATGAAAAAACGGCTCAAAGAAATCGAGGAGGAGGCGGG

AGCTCTGAGGGAGATGCAAGCAAAGGTAGAAAAGGAGATGGGAGCAGTACAGGAT

AGCAGTAGCACGTCCGCGACTCAGGCGGAGAAGGAGGAAGTGGACTCCCGGAGTAT

ATACGTGGGCAACGTAGATTACGCCTGCACTCCCGAGGAGGTGCAGCAGCATTTCCA

GAGTTGTGGAACTGTGAACAGGGTAACCATCCTTACAGATAAATTCGGTCAACCGAA

GGGCTTTGCCTATGTGGAGTTTGTGGAAATAGACGCCGTGCAGAACGCCCTGCTTCT

CAATGAGACTGAGCTGCATGGACGGCAGTTGAAGGTCTCCGCCAAAAGGACTAATAT

ACCCGGAATGAAGCAAGAATTCAAGCTTATGCCAAAAAAGAAAAGAAAGGTGGGC

GGCAAGCTTGGTACCATGCTGCCTCCACTGGAGCGGCTGACCCTGGACGGCGGCGG

TACCGTGAGCAAGGGCGAGGAGCTGTTCACCGGGGTGGTGCCCATCCTGGTCGAGC

TGGACGGCGACGTAAACGGCCACAAGTTCAGCGTGTCCGGCGAGGGCGAGGGCGA

TGCCACCTACGGCAAGCTGACCCTGAAGTTCATCTGCACCACCGGCAAGCTGCCCGT

GCCCTGGCCCACCCTCGTGACCACCCTGACCTACGGCGTGCAGTGCTTCAGCCGCTA

CCCCGACCACATGAAGCAGCACGACTTCTTCAAGTCCGCCATGCCCGAAGGCTACGT

CCAGGAGCGCACCATCTTCTTCAAGGACGACGGCAACTACAAGACCCGCGCCGAGG

TGAAGTTCGAGGGCGACACCCTGGTGAACCGCATCGAGCTGAAGGGCATCGACTTC

AAGGAGGACGGCAACATCCTGGGGCACAAGCTGGAGTACAACTACAACAGCCACA

ACGTCTATATCATGGCCGACAAGCAGAAGAACGGCATCAAGGTGAACTTCAAGATCC

GCCACAACATCGAGGACGGCAGCGTGCAGCTCGCCGACCACTACCAGCAGAACACC

CCCATCGGCGACGGCCCCGTGCTGCTGCCCGACAACCACTACCTGAGCACCCAGTC

CGCCCTGAGCAAAGACCCCAACGAGAAGCGCGATCACATGGTCCTGCTGGAGTTCG

TGACCGCCGCCGGGATCACTCTCGGCATGGACGAGCTGTACAAGTAACTCGAGCTTA

AG

SYG-NLS-NES-GFP protein

MGSYSSYGGYGGYSGYSGYGSYGSYSSYGSYSGYSGYGSYSSYGGYSGYGGYSGYGG

YGSYGSYGSYSGYGGYSSYSGYGSYGGYSGYSSYGGYGSYSSYSGYSSYSGYGSYSGY

GGYSSYGGYSSYGSYSGYGSYGSYSGYGSYSSYSGYSGYGSYGGYSSYSSYGGYGSYG

GYGGYSSYSSYSGYSSYGGYGGYSGYSGYGSREFSKDLEDMKKRLKEIEEEAGALREM

QAKVEKEMGAVQDSSSTSATQAEKEEVDSRSIYVGNVDYACTPEEVQQHFQSCGTVNR

VTILTDKFGQPKGFAYVEFVEIDAVQNALLLNETELHGRQLKVSAKRTNIPGMKQEFKL

MPKKKRKVGGKLGTMLPPLERLTLDGGGTVSKGEELFTGVVPILVELDGDVNGHKFSV

SGEGEGDATYGKLTLKFICTTGKLPVPWPTLVTTLTYGVQCFSRYPDHMKQHDFFKSA

MPEGYVQERTIFFKDDGNYKTRAEVKFEGDTLVNRIELKGIDFKEDGNILGHKLEYNYN

SHNVYIMADKQKNGIKVNFKIRHNIEDGSVQLADHYQQNTPIGDGPVLLPDNHYLSTQ

SALSKDPNEKRDHMVLLEFVTAAGITLGMDELYK*

[NLS (MPKKKRKVGG) or NES (MLPPLERLTLDGG)]

SYGQ-NLS-NES-GFP cDNA

GCTAGCCTCGAGTCTAGAATGGGATCCTATTCCCAGAGTTATGGCCAGGGCTACGGG

CAGGGATATAGCCAGGGATATAGCCAAGGGTACGGCCAGAGCTATGGACAAAGTTAC

AGTCAATCTTATGGTCAAAGTTATTCACAAGGCTACTCCCAGGGATACGGGCAGTCCT

ACTCCCAAAGCTACGGTCAGGGTTACTCCCAGGGATACGGACAAGGATACAGTCAA

GGCTACGGACAAGGCTACGGTCAGAGCTATGGTCAAAGCTACGGGCAAAGCTACTC

CCAGGGGTACGGTCAAGGATATTCTCAAAGTTACAGTCAGGGATCCTATGGCCAAAG

TTACGGACAGGGGTATTCCCAAGGATACAGCCAGTCTTATGGTCAAGGGTATGGTCA

ATCTTATTCTCAATCTTATTCTCAAGGCTACTCTCAGAGCTATAGTCAAGGTTACGGGC

AGTCCTATTCTCAAGGATACGGACAGGGATATTCACAGAGTTATGGACAGGGGTATTC

TCAAAGCTACGGTCAAAGTTATAGCCAAGGTTACGGCCAGAGCTATGGACAATCCTA

TAGCCAGGGATACGGACAGTCTTACTCTCAGTCTTATAGTCAGGGTTACAGCCAATCT

AGAGAATTCTCCAAGGATCTTGAGGACATGAAAAAACGGCTCAAAGAAATCGAGGA

GGAGGCGGGAGCTCTGAGGGAGATGCAAGCAAAGGTAGAAAAGGAGATGGGAGCA

GTACAGGATAGCAGTAGCACGTCCGCGACTCAGGCGGAGAAGGAGGAAGTGGACTC

CCGGAGTATATACGTGGGCAACGTAGATTACGCCTGCACTCCCGAGGAGGTGCAGCA

GCATTTCCAGAGTTGTGGAACTGTGAACAGGGTAACCATCCTTACAGATAAATTCGG

TCAACCGAAGGGCTTTGCCTATGTGGAGTTTGTGGAAATAGACGCCGTGCAGAACG

CCCTGCTTCTCAATGAGACTGAGCTGCATGGACGGCAGTTGAAGGTCTCCGCCAAA

AGGACTAATATACCCGGAATGAAGCAAGAATTCAAGCTTATGCCAAAAAAGAAAAG

AAAGGTGGGCGGCAAGCTTGGTACCATGCTGCCTCCACTGGAGCGGCTGACCCTGG

ACGGCGGCGGTACCGTGAGCAAGGGCGAGGAGCTGTTCACCGGGGTGGTGCCCATC

CTGGTCGAGCTGGACGGCGACGTAAACGGCCACAAGTTCAGCGTGTCCGGCGAGGG

CGAGGGCGATGCCACCTACGGCAAGCTGACCCTGAAGTTCATCTGCACCACCGGCA

AGCTGCCCGTGCCCTGGCCCACCCTCGTGACCACCCTGACCTACGGCGTGCAGTGCT
TCAGCCGCTACCCCGACCACATGAAGCAGCACGACTTCTTCAAGTCCGCCATGCCCG
AAGGCTACGTCCAGGAGCGCACCATCTTCTTCAAGGACGACGGCAACTACAAGACC
CGCGCCGAGGTGAAGTTCGAGGGCGACACCCTGGTGAACCGCATCGAGCTGAAGG
GCATCGACTTCAAGGAGGACGGCAACATCCTGGGGCACAAGCTGGAGTACAACTAC
AACAGCCACAACGTCTATATCATGGCCGACAAGCAGAAGAACGGCATCAAGGTGAA
CTTCAAGATCCGCCACAACATCGAGGACGGCAGCGTGCAGCTCGCCGACCACTACC
AGCAGAACACCCCCATCGGCGACGGCCCCGTGCTGCTGCCCGACAACCACTACCTG
AGCACCCAGTCCGCCCTGAGCAAAGACCCCAACGAGAAGCGCGATCACATGGTCCT
GCTGGAGTTCGTGACCGCCGCCGGGATCACTCTCGGCATGGACGAGCTGTACAAGT
AACTCGAGCTTAAG

SYGQ-NLS-NES-GFP protein

MGSYSQSYGQGYGQGYSQGYSQGYGQSYGQSYSQSYGQSYSQGYSQGYGQSYSQSY
GQGYSQGYGQGYSQGYGQGYGQSYGQSYGQSYSQGYGQGYSQSYSQGSYGQSYGQG
YSQGYSQSYGQGYGQSYSQSYSQGYSQSYSQGYGQSYSQGYGQGYSQSYGQGYSQSY
GQSYSQGYGQSYGQSYSQGYGQSYSQSYSQGYSQSREFSKDLEDMKKRLKEIEEEAGA
LREMQAKVEKEMGAVQDSSSTSATQAEKEEVDSRSIYVGNVDYACTPEEVQQHFQSCG
TVNRVTILTDKFGQPKGFAYVEFVEIDAVQNALLLNETELHGRQLKVSAKRTNIPGMKQ
EFKLMPKKKRKVGGKLGTMLPPLERLTLDGGGTVSKGEELFTGVVPILVELDGDVNGH
KFSVSGEGEGDATYGKLTLKFICTTGKLPVPWPTLVTTLTYGVQCFSRYPDHMKQHDFF
KSAMPEGYVQERTIFFKDDGNYKTRAEVKFEGDTLVNRIELKGIDFKEDGNILGHKLEY
NYNSHNVYIMADKQKNGIKVNFKIRHNIEDGSVQLADHYQQNTPIGDGPVLLPDNHYL
STQSALSKDPNEKRDHMVLLEFVTAAGITLGMDELYK*

SYGQ/N-NLS-NES-GFP cDNA

GCTAGCCTCGAGTTACCATGTCCTACTCCCAATCTTACGGCAATGGTTATGGACAGGG

ATATAGTAACGGTTACAGTCAAGGTTATGGGAATAGTTATGGGCAGAGCTACAGCAA

CTCTTACGGACAAAGCTATTCCAATGGTTACTCTCAGGGATACGGCAACTCCTACTCA

CAATCCTACGGCAATGGGTACTCTCAGGGTTACGGTAACGGCTACAGTCAAGGGTAT

GGGAATGGATACGGCCAGAGCTATGGAAACTCTTATGGACAAAGCTACTCAAATGGA

TACGGCCAGGGTTATTCTAACTCATATTCTCAAGGGTATGGTAATTCATATGGTCAGGG

GTATAGCAACGGATACTCCCAAAGTTACGGTAATGGCTACGGACAGTCCTATTCTAAC

TCATACAGTCAAGGTTATTCAAATAGTTACTCACAGGGATATGGCAACAGCTATTCTC

AAGGCTATGGAAATGGATACAGTCAGAGCTATGGAAACGGATATTCTCAAAGCTATG

GAAATAGCTACAGCCAGGGCTATGGCAACAGCTATGGCCAAAGCTATTCAAATGGAT

ATGGACAGAGCTATTCCAACTCCTATTCACAAGGCTATTCTAATTCTAGAGAATTCTC

CAAGGATCTTGAGGACATGAAAAAACGGCTCAAAGAAATCGAGGAGGAGGCGGGA

GCTCTGAGGGAGATGCAAGCAAAGGTAGAAAAGGAGATGGGAGCAGTACAGGATA

GCAGTAGCACGTCCGCGACTCAGGCGGAGAAGGAGGAAGTGGACTCCCGGAGTATA

TACGTGGGCAACGTAGATTACGCCTGCACTCCCGAGGAGGTGCAGCAGCATTTCCAG

AGTTGTGGAACTGTGAACAGGGTAACCATCCTTACAGATAAATTCGGTCAACCGAAG

GGCTTTGCCTATGTGGAGTTTGTGGAAATAGACGCCGTGCAGAACGCCCTGCTTCTC

AATGAGACTGAGCTGCATGGACGGCAGTTGAAGGTCTCCGCCAAAAGGACTAATATA

CCCGGAATGAAGCAAGAATTCAAGCTTATGCCAAAAAAGAAAAGAAAGGTGGGCG

GCAAGCTTGGTACCATGCTGCCTCCACTGGAGCGGCTGACCCTGGACGGCGGCGGT

ACCGTGAGCAAGGGCGAGGAGCTGTTCACCGGGGTGGTGCCCATCCTGGTCGAGCT

GGACGGCGACGTAAACGGCCACAAGTTCAGCGTGTCCGGCGAGGGCGAGGGCGAT

GCCACCTACGGCAAGCTGACCCTGAAGTTCATCTGCACCACCGGCAAGCTGCCCGT

GCCCTGGCCCACCCTCGTGACCACCCTGACCTACGGCGTGCAGTGCTTCAGCCGCTA

CCCCGACCACATGAAGCAGCACGACTTCTTCAAGTCCGCCATGCCCGAAGGCTACGT

CCAGGAGCGCACCATCTTCTTCAAGGACGACGGCAACTACAAGACCCGCGCCGAGG

TGAAGTTCGAGGGCGACACCCTGGTGAACCGCATCGAGCTGAAGGGCATCGACTTC

AAGGAGGACGGCAACATCCTGGGGCACAAGCTGGAGTACAACTACAACAGCCACA

ACGTCTATATCATGGCCGACAAGCAGAAGAACGGCATCAAGGTGAACTTCAAGATCC

GCCACAACATCGAGGACGGCAGCGTGCAGCTCGCCGACCACTACCAGCAGAACACC

CCCATCGGCGACGGCCCCGTGCTGCTGCCCGACAACCACTACCTGAGCACCCAGTC

CGCCCTGAGCAAAGACCCCAACGAGAAGCGCGATCACATGGTCCTGCTGGAGTTCG

TGACCGCCGCCGGGATCACTCTCGGCATGGACGAGCTGTACAAGTAACTCGAGCTTA

AG

SYGQ/N-NLS-NES-GFP protein

MSYSQSYGNGYGQGYSNGYSQGYGNSYGQSYSNSYGQSYSNGYSQGYGNSYSQSYG

NGYSQGYGNGYSQGYGNGYGQSYGNSYGQSYSNGYGQGYSNSYSQGYGNSYGQGYS

NGYSQSYGNGYGQSYSNSYSQGYSNSYSQGYGNSYSQGYGNGYSQSYGNGYSQSYG

NSYSQGYGNSYGQSYSNGYGQSYSNSYSQGYSNSREFSKDLEDMKKRLKEIEEEAGAL

REMQAKVEKEMGAVQDSSSTSATQAEKEEVDSRSIYVGNVDYACTPEEVQQHFQSCGT

VNRVTILTDKFGQPKGFAYVEFVEIDAVQNALLLNETELHGRQLKVSAKRTNIPGMKQE

FKLMPKKKRKVGGKLGTMLPPLERLTLDGGGTVSKGEELFTGVVPILVELDGDVNGHK

FSVSGEGEGDATYGKLTLKFICTTGKLPVPWPTLVTTLTYGVQCFSRYPDHMKQHDFFK

SAMPEGYVQERTIFFKDDGNYKTRAEVKFEGDTLVNRIELKGIDFKEDGNILGHKLEYN

YNSHNVYIMADKQKNGIKVNFKIRHNIEDGSVQLADHYQQNTPIGDGPVLLPDNHYLS

TQSALSKDPNEKRDHMVLLEFVTAAGITLGMDELYK*

sPFD-NLS-NES-GFP cDNA

GCTAGCCTCGAGTCTAGAATGTCACAACAGTATAACCAAAATAACCTTTATCAACAA
GGGCAACAACAGAATAACGGGGAGCAATCCTTCTGGTACCAACAAAACAATAATTT
GCAGCAACAAGGTAACTATCAACAGTACAATTACACTAATGGAAATAACAACCAGAC
ATCACAAATATCCCAAGGGCAACAGAATGGAGGCAATCAAAATCAGAATAACCGAC

AACAAAACCAGAACCAGAATACCGCACCAAACTCAACTTCTACTAGCACCAACGGA
TACGGCGCTTCTGGGCACGGTCGGAGTACAACGTCTTATGGGGTGCAAGATCACTCT
GGAGCACGCATTGAAAGTGCGGCTAGCATGTCTAGAGAATTCTCCAAGGATCTTGAG
GACATGAAAAAACGGCTCAAAGAAATCGAGGAGGAGGCGGGAGCTCTGAGGGAGA
TGCAAGCAAAGGTAGAAAAGGAGATGGGAGCAGTACAGGATAGCAGTAGCACGTCC
GCGACTCAGGCGGAGAAGGAGGAAGTGGACTCCCGGAGTATATACGTGGGCAACGT
AGATTACGCCTGCACTCCCGAGGAGGTGCAGCAGCATTTCCAGAGTTGTGGAACTGT
GAACAGGGTAACCATCCTTACAGATAAATTCGGTCAACCGAAGGGCTTTGCCTATGT
GGAGTTTGTGGAAATAGACGCCGTGCAGAACGCCCTGCTTCTCAATGAGACTGAGC
TGCATGGACGGCAGTTGAAGGTCTCCGCCAAAAGGACTAATATACCCGGAATGAAGC
AAGAATTCAAGCTTATGCCAAAAAAGAAAAGAAAGGTGGGCGGCAAGCTTGGTACC
ATGCTGCCTCCACTGGAGCGGCTGACCCTGGACGGCGGCGGTACCGTGAGCAAGGG
CGAGGAGCTGTTCACCGGGGTGGTGCCCATCCTGGTCGAGCTGGACGGCGACGTAA
ACGGCCACAAGTTCAGCGTGTCCGGCGAGGGCGAGGGCGATGCCACCTACGGCAAG
CTGACCCTGAAGTTCATCTGCACCACCGGCAAGCTGCCCGTGCCCTGGCCCACCCTC
GTGACCACCCTGACCTACGGCGTGCAGTGCTTCAGCCGCTACCCCGACCACATGAA
GCAGCACGACTTCTTCAAGTCCGCCATGCCCGAAGGCTACGTCCAGGAGCGCACCA
TCTTCTTCAAGGACGACGGCAACTACAAGACCCGCGCCGAGGTGAAGTTCGAGGGC
GACACCCTGGTGAACCGCATCGAGCTGAAGGGCATCGACTTCAAGGAGGACGGCAA
CATCCTGGGGCACAAGCTGGAGTACAACTACAACAGCCACAACGTCTATATCATGGC
CGACAAGCAGAAGAACGGCATCAAGGTGAACTTCAAGATCCGCCACAACATCGAGG
ACGGCAGCGTGCAGCTCGCCGACCACTACCAGCAGAACACCCCCATCGGCGACGGC
CCCGTGCTGCTGCCCGACAACCACTACCTGAGCACCCAGTCCGCCCTGAGCAAAGA
CCCCAACGAGAAGCGCGATCACATGGTCCTGCTGGAGTTCGTGACCGCCGCCGGGA
TCACTCTCGGCATGGACGAGCTGTACAAGTAACTCGAGCTTAAG

sPFD-NLS-NES-GFP protein

ASLESRMSQQYNQNNLYQQGQQQNNGEQSFWYQQNNNLQQQGNYQQYNYTNGNNN

QTSQISQGQQNGGNQNQNNRQQNQNQNTAPNSTSTSTNGYGASGHGRSTTSYGVQDH

SGARIESAASMSREFSKDLEDMKKRLKEIEEEAGALREMQAKVEKEMGAVQDSSSTSA

TQAEKEEVDSRSIYVGNVDYACTPEEVQQHFQSCGTVNRVTILTDKFGQPKGFAYVEFV

EIDAVQNALLLNETELHGRQLKVSAKRTNIPGMKQEFKLMPKKKRKVGGKLGTMLPPL

ERLTLDGGGTVSKGEELFTGVVPILVELDGDVNGHKFSVSGEGEGDATYGKLTLKFICT

TGKLPVPWPTLVTTLTYGVQCFSRYPDHMKQHDFFKSAMPEGYVQERTIFFKDDGNYK

TRAEVKFEGDTLVNRIELKGIDFKEDGNILGHKLEYNYNSHNVYIMADKQKNGIKVNF

KIRHNIEDGSVQLADHYQQNTPIGDGPVLLPDNHYLSTQSALSKDPNEKRDHMVLLEF

VTAAGITLGMDELYK*

cPFD-NLS-NES-GFP cDNA

GCTAGCCTCGAGTCTAGAATGAGTCAGGCGGCTTCTACAAAGCAATCTACGGAGACC

CAAAATGGAGCACTTAATCAAACGCAGGACCAAAACCATCACCAGACTCCGGTAGG

TCGCAATAACCAGAATGGCACCCAAAACCCATACAATTCAGAACAGCCCAATCAGAA

TAATTGGAATACGAGGAATCAATCAAATAACAGCGCACAAAATCAGCAGCCCCAGCA

GGATAACCAAAATAATACACGCGGGAATCAGCAGCAGGAACCCCAGCAAGCCAGCG

GCACCTCTTTGGCTATGAATCAGCATACTAAGTTGAATAACGAAAACAATTCCCAGGA

TTTCTTGCAGCAAATGTGGTCTAGAGAATTCTCCAAGGATCTTGAGGACATGAAAAA

ACGGCTCAAAGAAATCGAGGAGGAGGCGGGAGCTCTGAGGGAGATGCAAGCAAAG

GTAGAAAAGGAGATGGGAGCAGTACAGGATAGCAGTAGCACGTCCGCGACTCAGGC

GGAGAAGGAGGAAGTGGACTCCCGGAGTATATACGTGGGCAACGTAGATTACGCCT

GCACTCCCGAGGAGGTGCAGCAGCATTTCCAGAGTTGTGGAACTGTGAACAGGGTA

ACCATCCTTACAGATAAATTCGGTCAACCGAAGGGCTTTGCCTATGTGGAGTTTGTGG

AAATAGACGCCGTGCAGAACGCCCTGCTTCTCAATGAGACTGAGCTGCATGGACGG

CAGTTGAAGGTCTCCGCCAAAAGGACTAATATACCCGGAATGAAGCAAGAATTCAA

GCTTATGCCAAAAAAGAAAAGAAAGGTGGGCGGCAAGCTTGGTACCATGCTGCCTC

CACTGGAGCGGCTGACCCTGGACGGCGGCGGTACCGTGAGCAAGGGCGAGGAGCT

GTTCACCGGGGTGGTGCCCATCCTGGTCGAGCTGGACGGCGACGTAAACGGCCACA

AGTTCAGCGTGTCCGGCGAGGGCGAGGGCGATGCCACCTACGGCAAGCTGACCCTG

AAGTTCATCTGCACCACCGGCAAGCTGCCCGTGCCCTGGCCCACCCTCGTGACCACC

CTGACCTACGGCGTGCAGTGCTTCAGCCGCTACCCCGACCACATGAAGCAGCACGA

CTTCTTCAAGTCCGCCATGCCCGAAGGCTACGTCCAGGAGCGCACCATCTTCTTCAA

GGACGACGGCAACTACAAGACCCGCGCCGAGGTGAAGTTCGAGGGCGACACCCTG

GTGAACCGCATCGAGCTGAAGGGCATCGACTTCAAGGAGGACGGCAACATCCTGGG

GCACAAGCTGGAGTACAACTACAACAGCCACAACGTCTATATCATGGCCGACAAGC

AGAAGAACGGCATCAAGGTGAACTTCAAGATCCGCCACAACATCGAGGACGGCAGC

GTGCAGCTCGCCGACCACTACCAGCAGAACACCCCCATCGGCGACGGCCCCGTGCT

GCTGCCCGACAACCACTACCTGAGCACCCAGTCCGCCCTGAGCAAAGACCCCAACG

AGAAGCGCGATCACATGGTCCTGCTGGAGTTCGTGACCGCCGCCGGGATCACTCTCG

GCATGGACGAGCTGTACAAGTAACTCGAGCTTAAG

cPFD-NLS-NES-GFP protein

ASLESRMSQAASTKQSTETQNGALNQTQDQNHHQTPVGRNNQNGTQNPYNSEQPNQN

NWNTRNQSNNSAQNQQPQQDNQNNTRGNQQQEPQQASGTSLAMNQHTKLNNENNS

QDFLQQMWSREFSKDLEDMKKRLKEIEEEAGALREMQAKVEKEMGAVQDSSSTSATQ

AEKEEVDSRSIYVGNVDYACTPEEVQQHFQSCGTVNRVTILTDKFGQPKGFAYVEFVEI

DAVQNALLLNETELHGRQLKVSAKRTNIPGMKQEFKLMPKKKRKVGGKLGTMLPPLE

RLTLDGGGTVSKGEELFTGVVPILVELDGDVNGHKFSVSGEGEGDATYGKLTLKFICTT

GKLPVPWPTLVTTLTYGVQCFSRYPDHMKQHDFFKSAMPEGYVQERTIFFKDDGNYKT

RAEVKFEGDTLVNRIELKGIDFKEDGNILGHKLEYNYNSHNVYIMADKQKNGIKVNFKI

RHNIEDGSVQLADHYQQNTPIGDGPVLLPDNHYLSTQSALSKDPNEKRDHMVLLEFVT

AAGITLGMDELYK*
